# Supplementary material for: Effect of Post-Harvest LED and UV Light Irradiation on the Accumulation of Flavonoids and Limonoids in the Segments of Newhall Navel Oranges (Citrus sinensis Osbeck)
Source: Molecules. 2019 May 6;24(9):1755. doi: 10.3390/molecules24091755 (PMC6540038; doi:10.3390/molecules24091755)
Supplement: Supplementary file 1 [file molecules-24-01755-s001.pdf]

Supporting Information

Effect of postharvest visible and UV light irradiation on flavonoid and limonoid accumulation in the segment of Newhall navel orange (Citrus sinensis Osbeck)

Shengyu Liu [1], Linping Hu [1], Dong Jiang [2] and Wanpeng Xi [1,3,*]

1 College of Horticulture and Landscape Architecture, Southwest University, Chongqing 400716, China

2 Citrus Research Institute, Chinese Academy of Agricultural Sciences, Chongqing 400712, China

3 Key Laboratory of Horticulture Science for Southern Mountainous Regions, Ministry of Education, Chongqing 400715, China

Table of contents page

Table S1. Compounds identification information from references
3

Figure S1. Base peak ion (BPI) chromatograms of methanolic extracts in Newhall navel orange fresh pulp under positive mode
6

Figure S2-S22. Mass chromatogram of 21 identified compounds in the positive mode at both low and high energy
6

Figure S23-43. ESI peak of 21 identified compounds in the positive mode
17

Table S1. Compounds identification information from references.

Peak order	Reference RT(min)	Reference[M+H]$^+$(error, ppm)	Reference diagnostic MS^2 ion (%)	Reference component name
1	3.44	595.1652(-0.84)	595.16486 (100), 457.11142 (46.50), 379.08057 (34.56), 325.06996 (33.73), 409.09124 (28.26), 477.11946 (2.90)	Apigenin-6,8-di-C-glucoside （Vicenin-2）
2	3.67	625.1758 (-0.83)	625.17525 (100), 487.12203 (51.8), 409.09114 (35.05), 355.08062 (34.18), 457.11121 (26.40), 367.08061 (23.74), 607.16445 (23.67)	Diosmetin 6,8-di-C-glucoside
3	3.77	741.2227 (-1.23)	433.11205 (100), 595.16528 (81.55), 271.05983 (52.55), 153.01842 (7.97), 163.03885 (2.91)	Rhoifolin-4'-O-glucoside
4	3.77	597.1802 (-2.02)	289.07025 (100), 435.12291 (28.40), 153.01842 (26.34), 451.11574 (22.49)	Isomer-eriocitrin (Neoeriocitrin)
5	3.84	625.1754 (-1.47)	625.17536(100), 285.07571 (54.38), 487.12206 (52.73), 355.08011 (40.40), 367.08135 (29.96), 457.11123 (29.63), 607.16421 (21.41)	Chysoeriol-6,8-di-C-glucoside （Stellarin-2）
6	3.86	743.2376 (-2.36)	273.07530 (100), 765.21903 (81.25), 153.01858 (21.56), 147.04434 (14.01), 435.12700 (10.94)	Narirutin-4'-glucoside
7	4.50	471.2010 (-0.75)	315.08606 (100), 425.19491 (53.99), 161.05999 (29.70), 273.07524 (18.90), 409.19922 (17.46),	Epilimonin

8	4.80	597.1813 (-0.20)	289.07061 (100), 153.01850 (26.36), 435.12806 (21.54), 163.03907 (12.65), 417.11780 (6.20),	Eriocitrin
9	5.79	581.1873 (1.41)	273.07607 (100), 329.10210 (59.52), 153.01889 (25.43), 493.13466 (18.64), 419.13413 (12.87)	Narirutin
10	6.40	609.1804 (-1.61)	301.07028(100), 463.12281(31.24), 286.04655(9.38), 258.05180(8.26)	Diosmin
11	6.55	611.1968 (-0.32)	303.08621 (100), 449.14391 (27.29), 153.01855 (18.68), 465.13882 (15.26), 177.05475 (13.36)	Hesperidin
12	6.91	611.1963 (-1.29)	303.08571 (100), 359.11168 (43.03), 153.01841 (34.19), 345.09633 (19.13)	Neohesperidin
13	7.13	515.2273 (-0.48)	515.22666 (100), 161.05971 (27.78), 303.08569 (22.54), 487.23222 (19.85), 469.22166 (16.15)	7α-Limonyl acetate
14	9.13	595.2026 (0.76)	287.09146 (100), 153.01867 (25.93), 389.12318 (22.15), 161.05989 (11.81), 433.14839 (9.66)	Didymin
15	10.54	373.1287 (1.51)	343.08196 (100), 373.12883 (52.57), 315.08609 (14.31), 357.09670 (11.62), 153.01851 (5.00), 181.01329 (4.48)	5,7,8,3',4'-Pentamethoxyflavone (Isosinensetin)

16	11.22	373.1287 (1.49)	373.12843 (100), 343.08136 (87.66), 312.09898 (55.95), 358.10339 (24.52), 153.01855 (5.48), 163.07527 (3.48)	5,6,7,3',4'-Pentamethoxyflavone (Sinensetin)
17	11.39	471.2007(-1.44)	343.11750 (100), 328.09362 (33.60), 161.05972 (0.30), 395.10899 (0.26), 425.19451 (0.22)	Limonin
18	12.14	403.1389 (0.31)	373.09213 (100), 403.13931 (37.25), 388.11528 (17.59), 327.08594 (11.24), 211.0238 (4.19), 183.02896 (2.73)	5,6,7,8,3',4'-Hexamethoxyflavone (Nobiletin)
19	12.23	343.1176 (-0.17)	313.07050 (100), 343.11699 (71.53), 282.08818 (65.23), 153.01835 (9.73), 181.01309 (4.87), 133.06490(4.36)	5,7,8,4'-Tetramethoxyflavone
20	12.80	433.1496 (0.58)	403.10285 (20.27), 433.14963 (11.43), 373.09222 (7.85), 404.10536 (4.61), 418.12492 (3.45), 385.09122(2.03)	3,5,6,7,8,3',4'-Heptamethoxyflavone
21	13.25	373.1289 (1.94)	343.08240 (100), 395.11067 (30.69), 373.12925 (27.96), 344.08502 (23.81), 297.07600 (10.44), 211.02418 (5.15)	5,6,7,8,4'-Pentamethoxyflavone (Tangeretin)

References

Zhao, X.J.; Xing, T.T.; Li, Y.F.; Jiao, B.N.; Jiang, D. Efficient analysis of phytochemical constituents in the peel of Chinese wild citrus Mangshanju (Citrus reticulata Blanco) by ultra high performance liquid chromatography–quadrupole time–of–flight–mass spectrometry. *J. Sep. Sci.* **2018**.

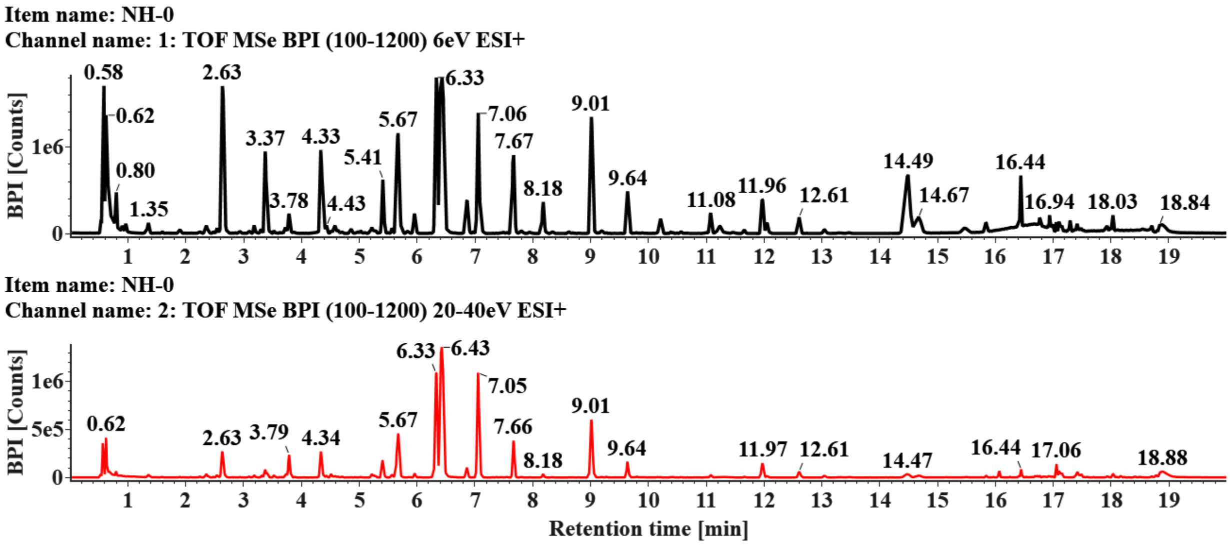

Figure S1. Base peak ion (BPI) chromatograms of methanolic extracts in Newhall navel orange fresh pulp under positive mode.

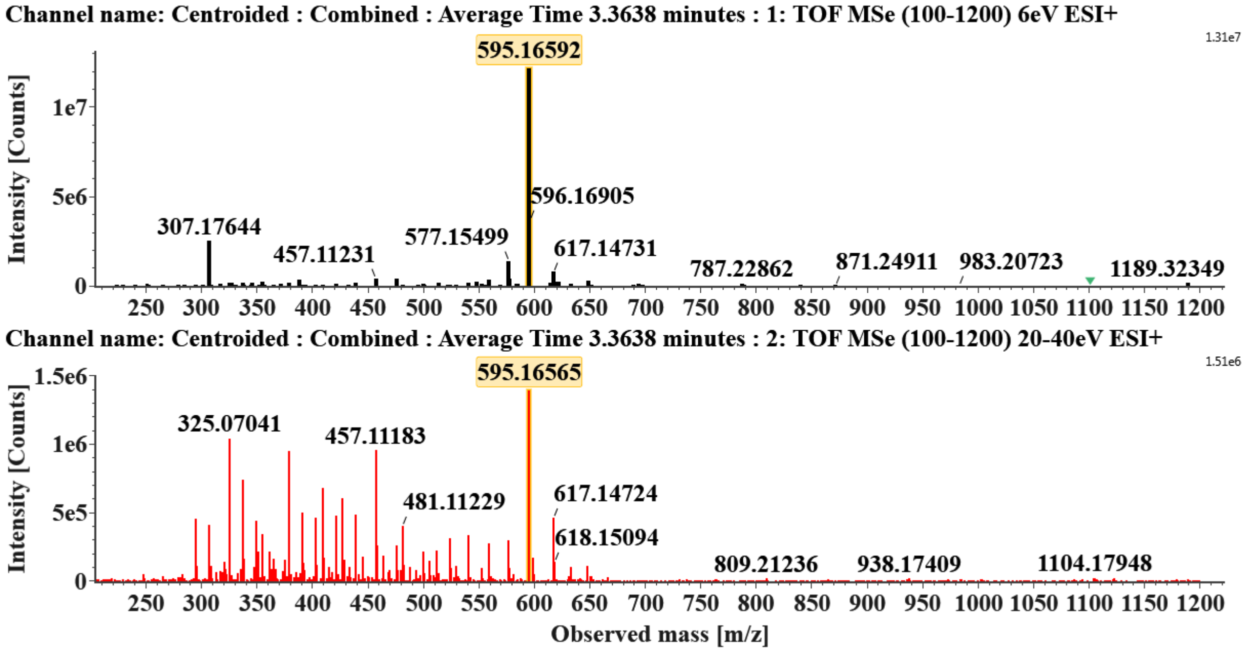

Figure. S2 The Mass chromatogram of Apigenin-6,8-di-C-glucoside (Vicenin-2) in positive mode: Low energy (Top), High energy (Below).

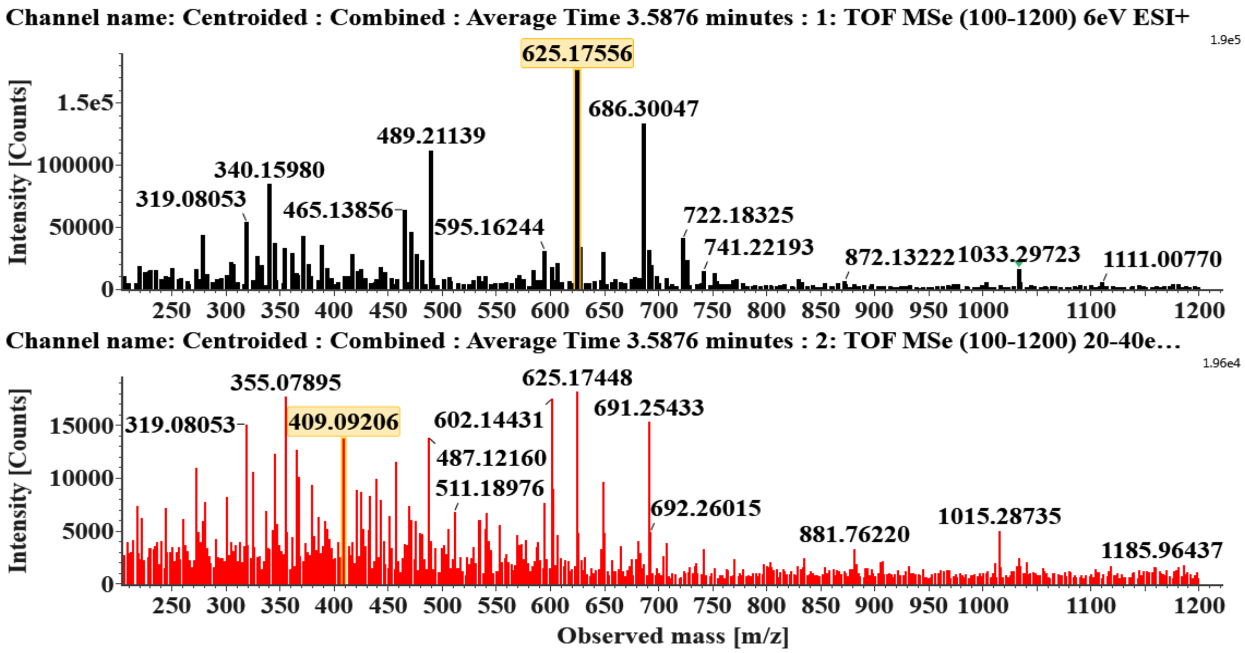

Figure.S3 The Mass chromatogram of Diosmetin 6,8-di-C-glucoside in positive mode: Low energy (Top), High energy (Below).

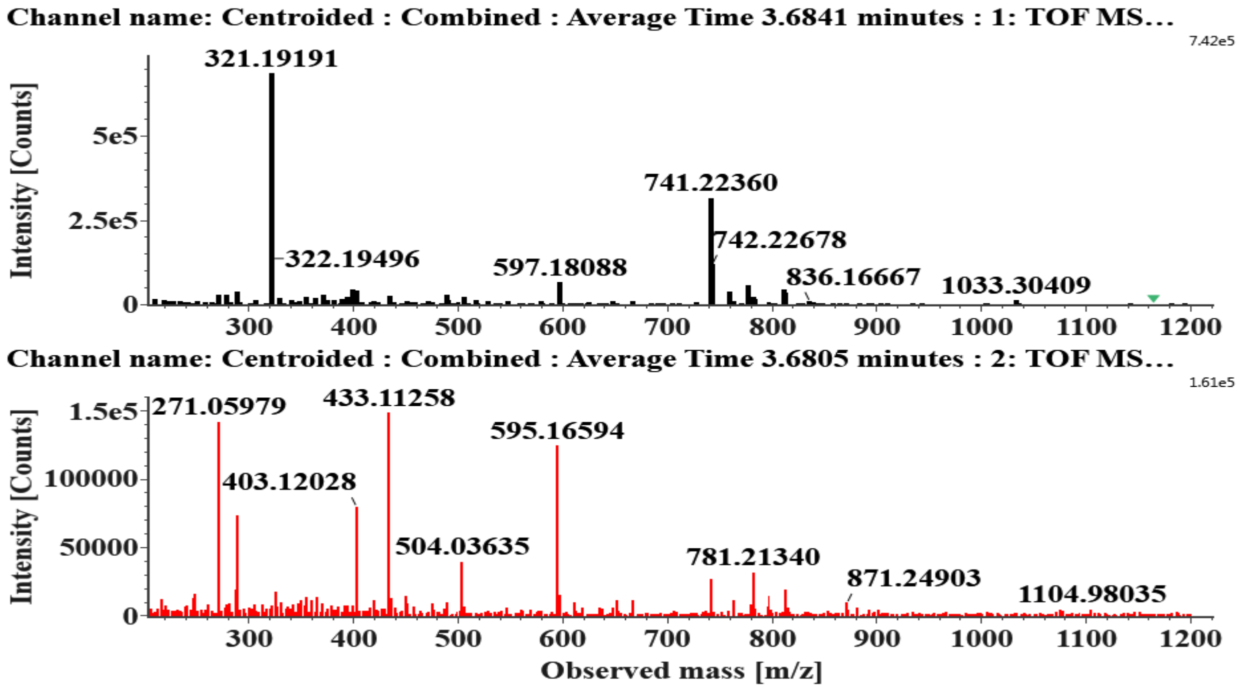

Figure.S4 The Mass chromatogram of Rhoifolin-4’-O-glucoside in positive mode: Low energy (Top), High energy (Below).

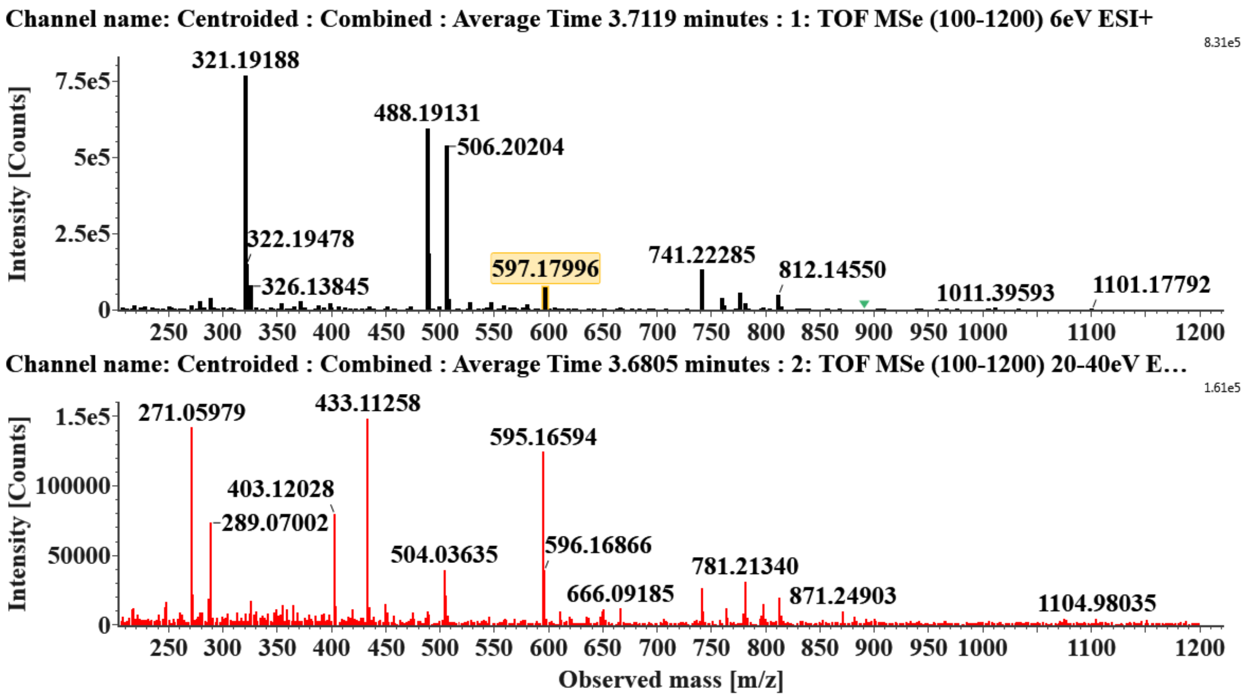

Figure.S5 The Mass chromatogram of Neoeriocitrin in positive mode: Low energy (Top), High energy (Below).

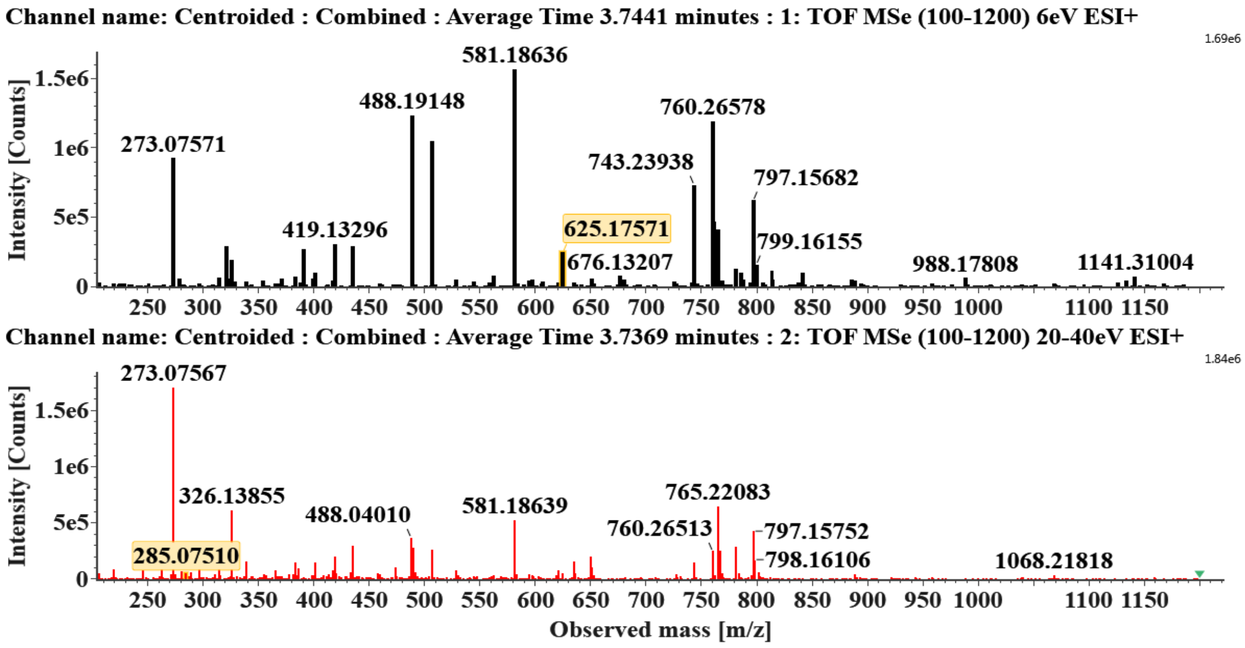

Figure.S6 The Mass chromatogram of Chysoeriol-6,8-di-C-glucoside (Stellarin-2) in positive mode: Low energy (Top), High energy (Below).

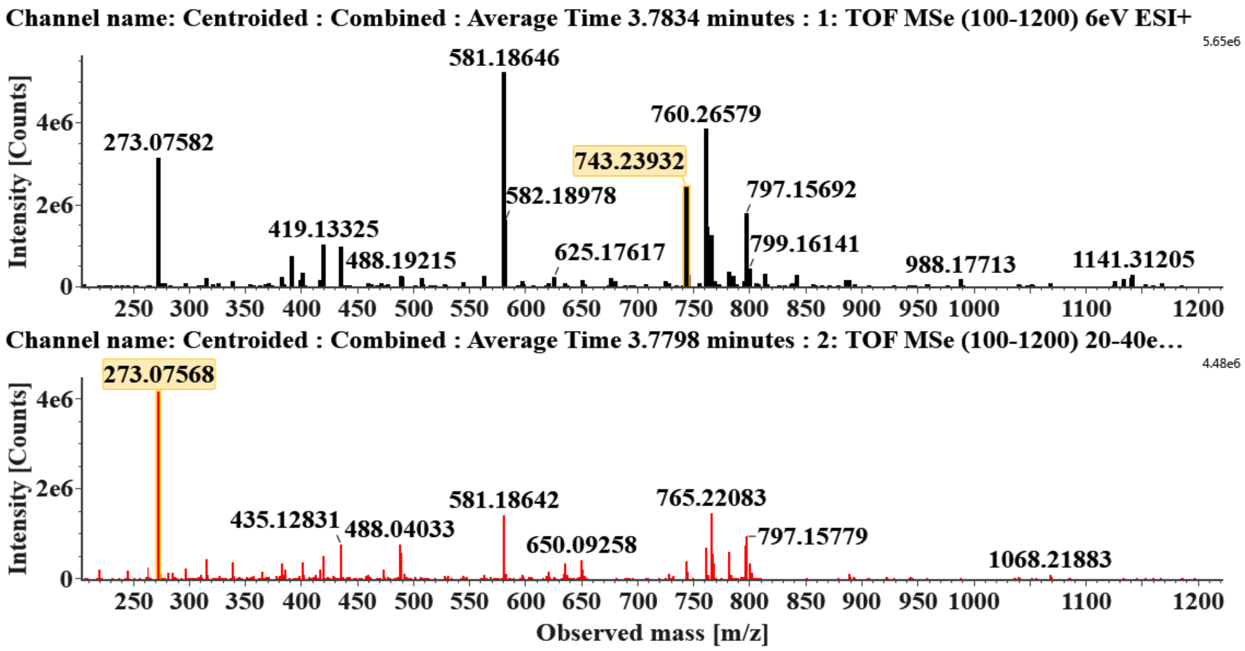

Figure.S7 The Mass chromatogram of Narirutin-4’-glucoside in positive mode: Low energy (Top), High energy (Below).

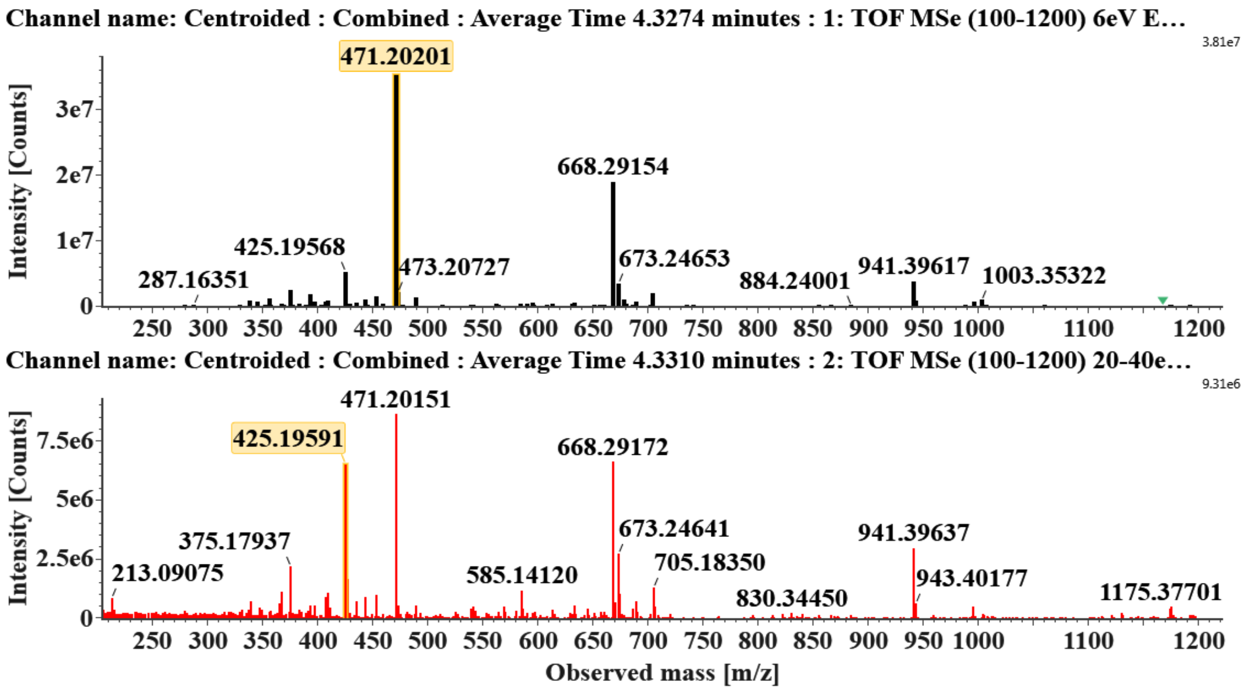

Figure.S8 The Mass chromatogram of Epilimonin in positive mode: Low energy (Top), High energy (Below).

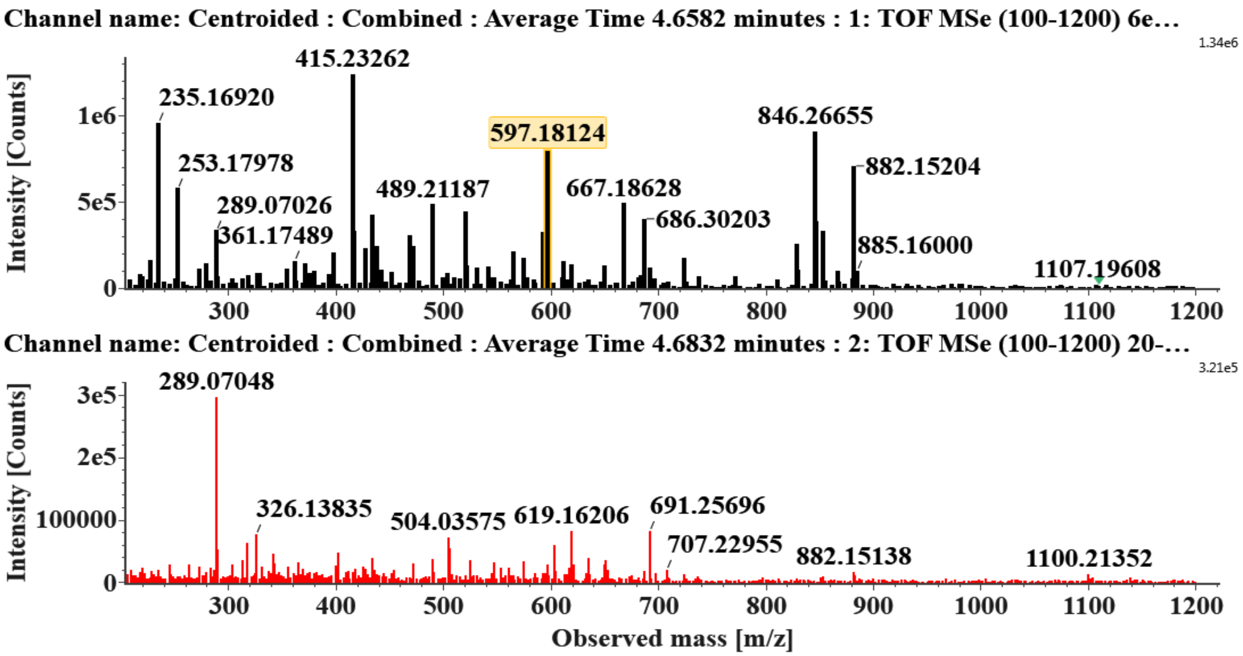

Figure.S9 The Mass chromatogram of Eriocitrin in positive mode: Low energy (Top), High energy (Below).

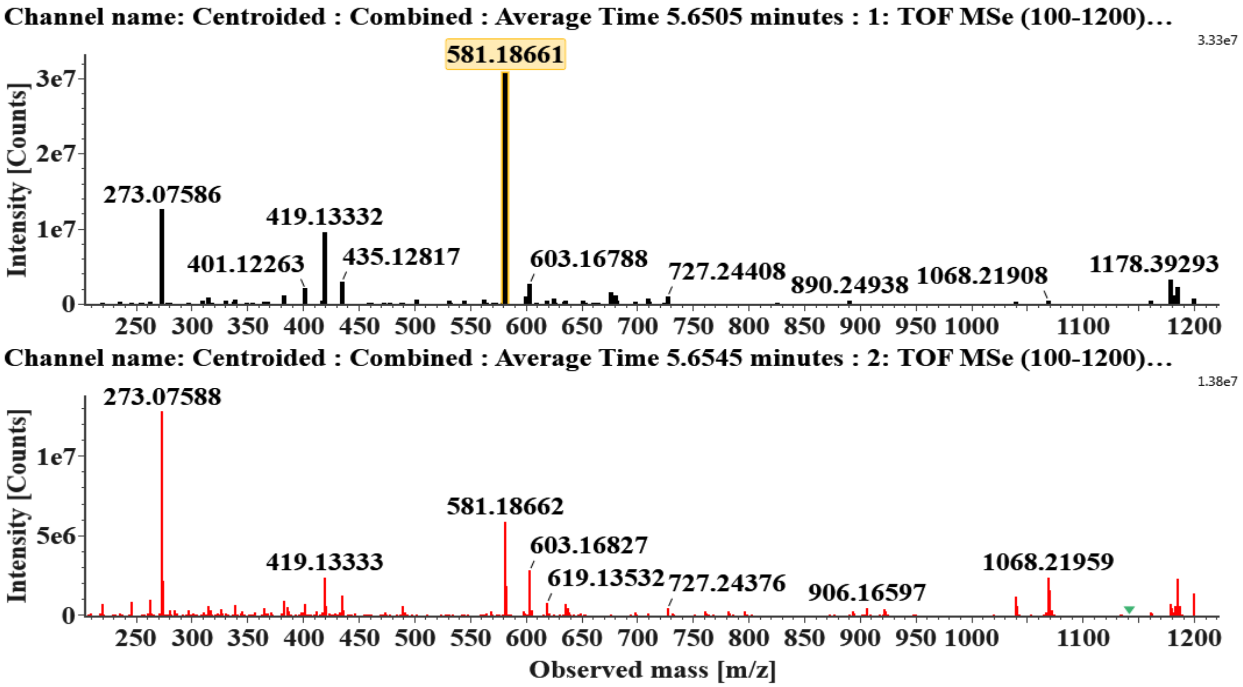

Figure.S10 The Mass chromatogram of Narirutin in positive mode: Low energy (Top), High energy (Below).

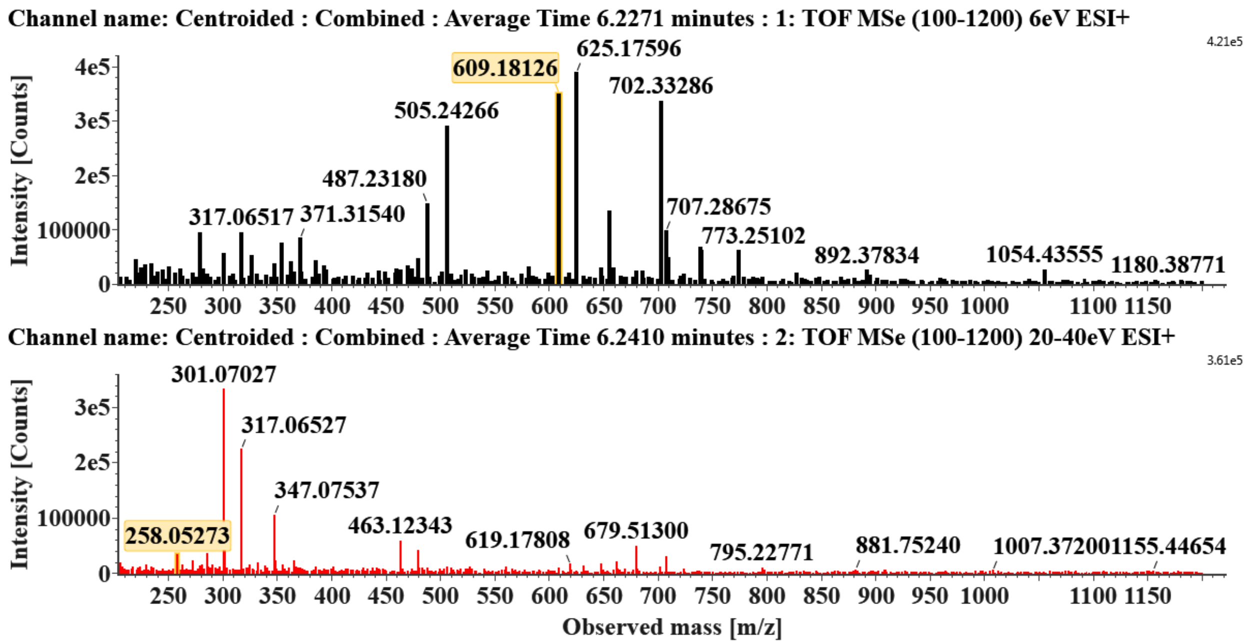

Figure.S11 The Mass chromatogram of Diosmin in positive mode: Low energy (Top), High energy (Below).

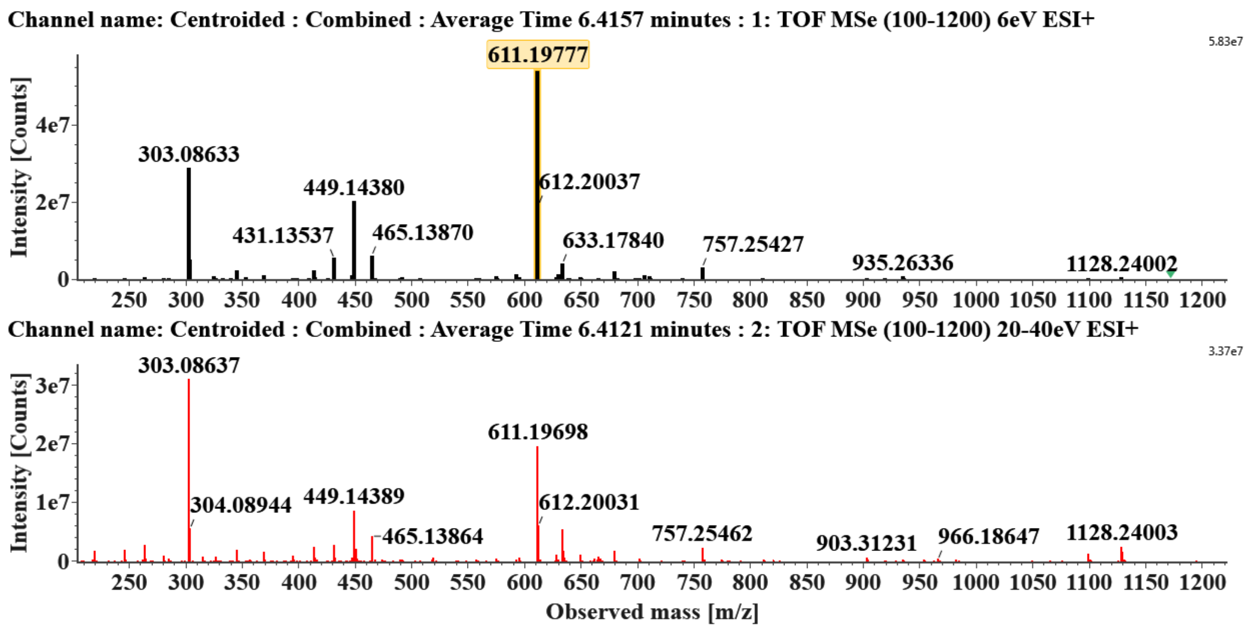

Figure.S12 The Mass chromatogram of Hesperidin in positive mode: Low energy (Top), High energy (Below).

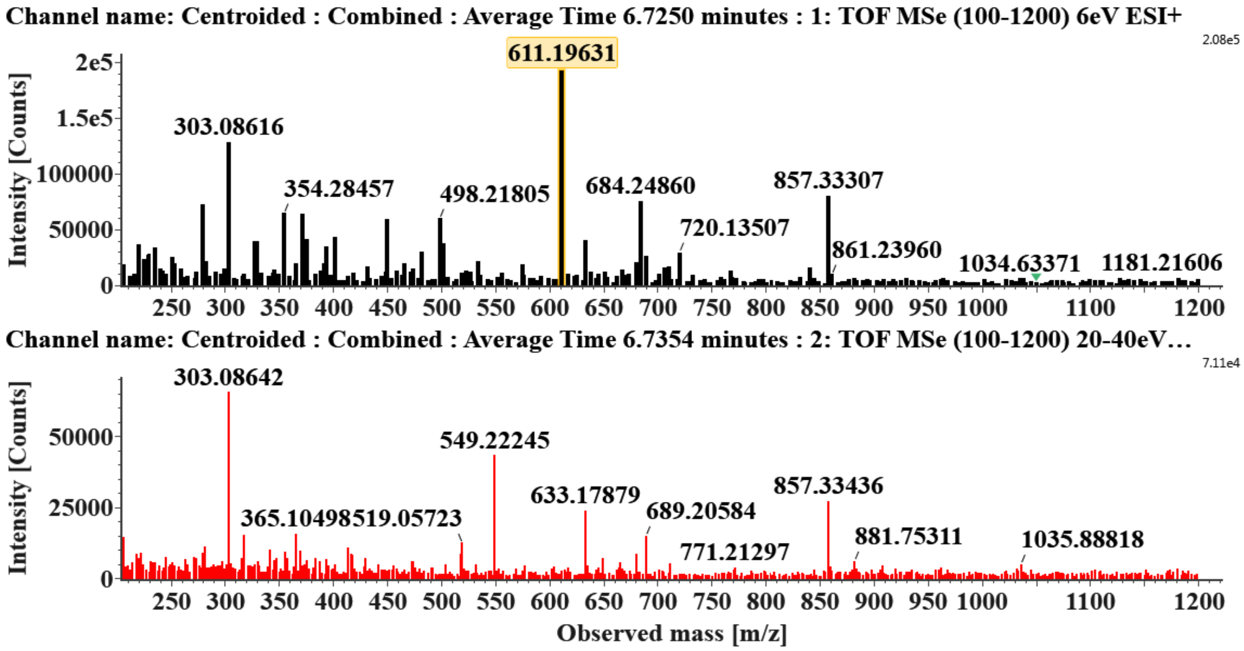

Figure.S13 The Mass chromatogram of Neohesperidin in positive mode: Low energy (Top), High energy (Below).

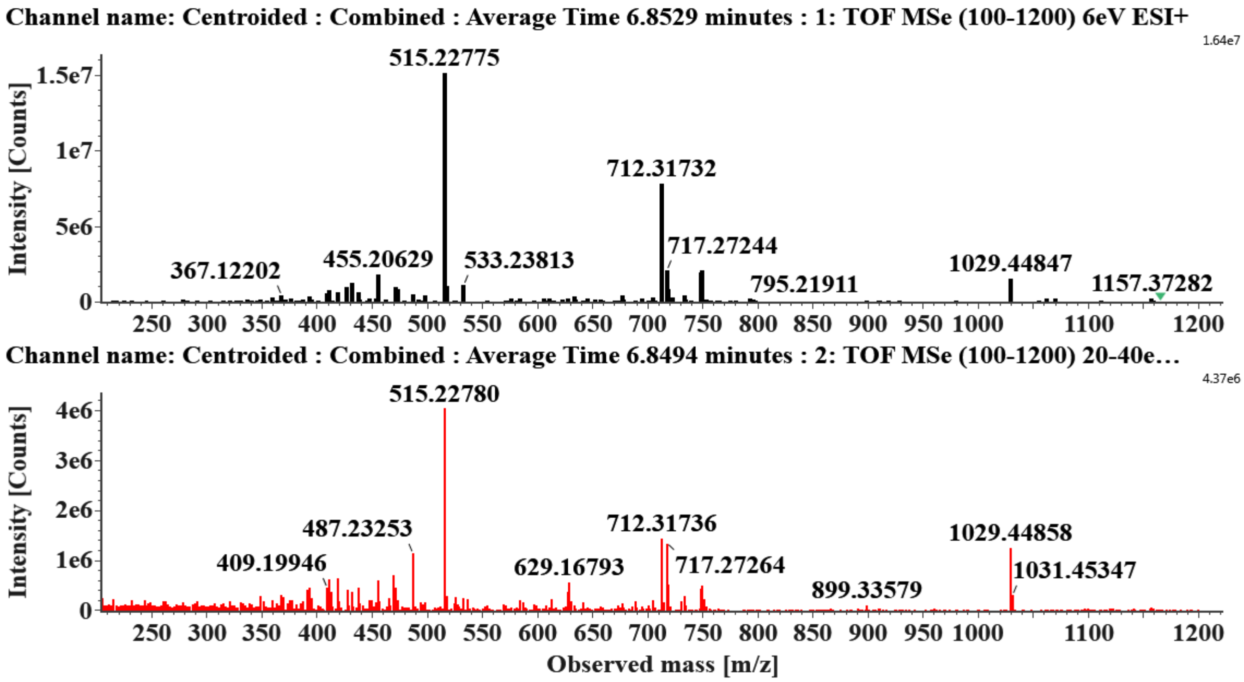

Figure.S14 The Mass chromatogram of 7a-Limonyl acetate in positive mode: Low energy (Top), High energy (Below).

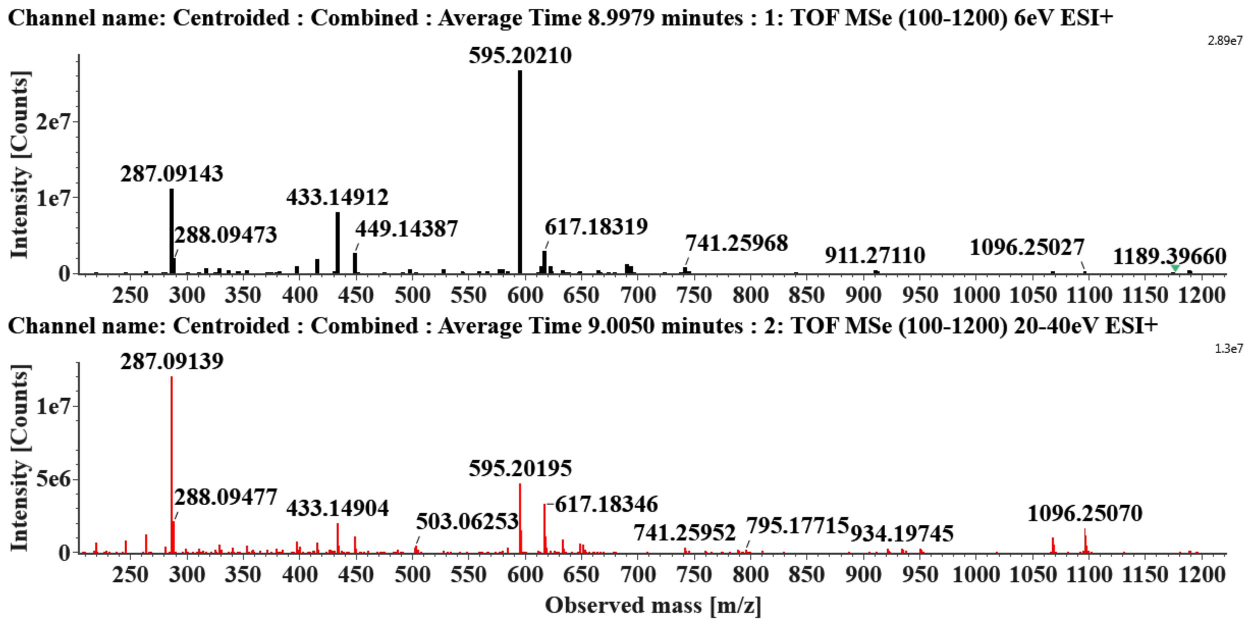

Figure.S15 The Mass chromatogram of Didymin in positive mode: Low energy (Top), High energy (Below).

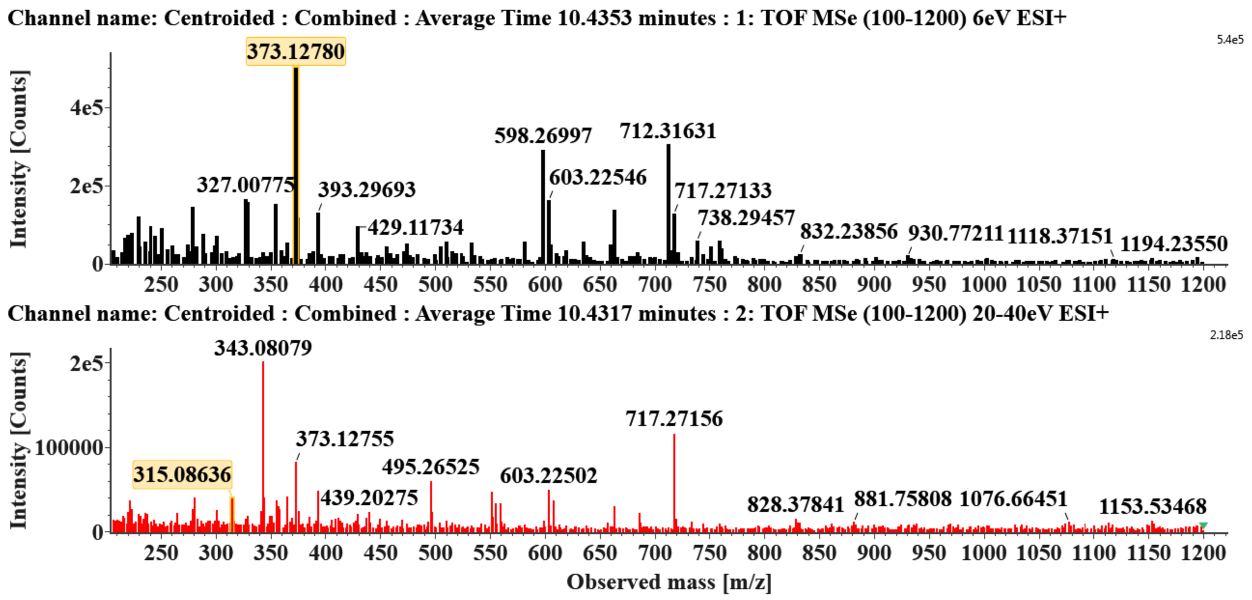

Figure.S16 The Mass chromatogram of 5,7,8,3',4'-Pentamethoxyflavone (Isosinensetin) in positive mode: Low energy (Top), High energy (Below).

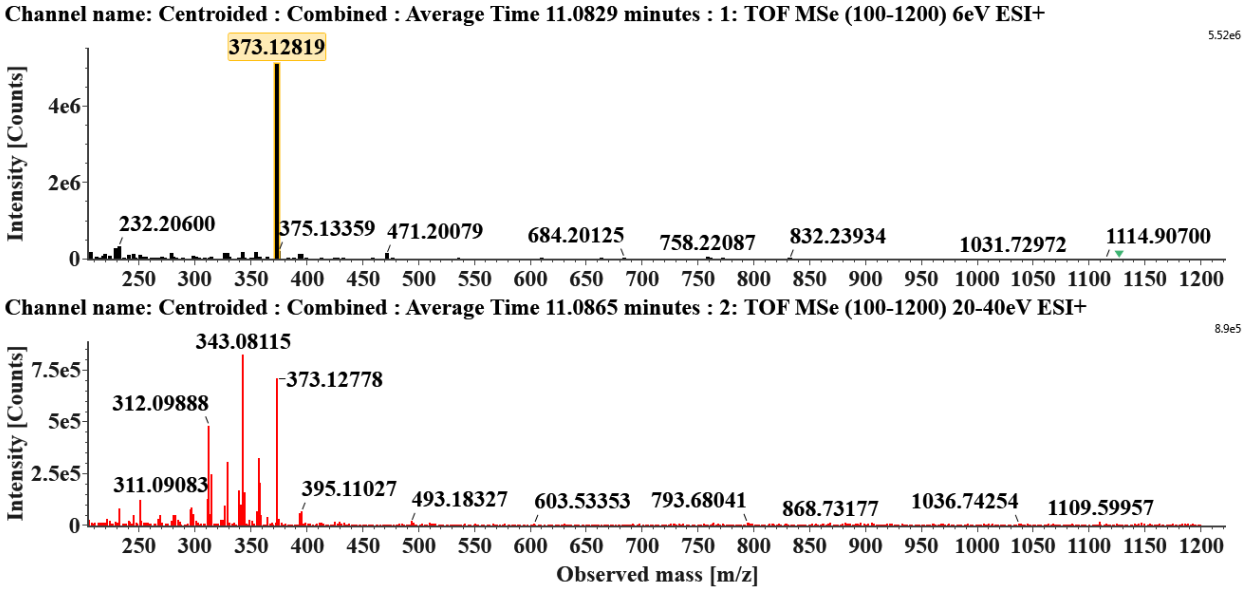

Figure.S17 The Mass chromatogram of 5,6,7,3',4'-Pentamethoxyflavone (Sinensetin) in positive mode: Low energy (Top), High energy (Below).

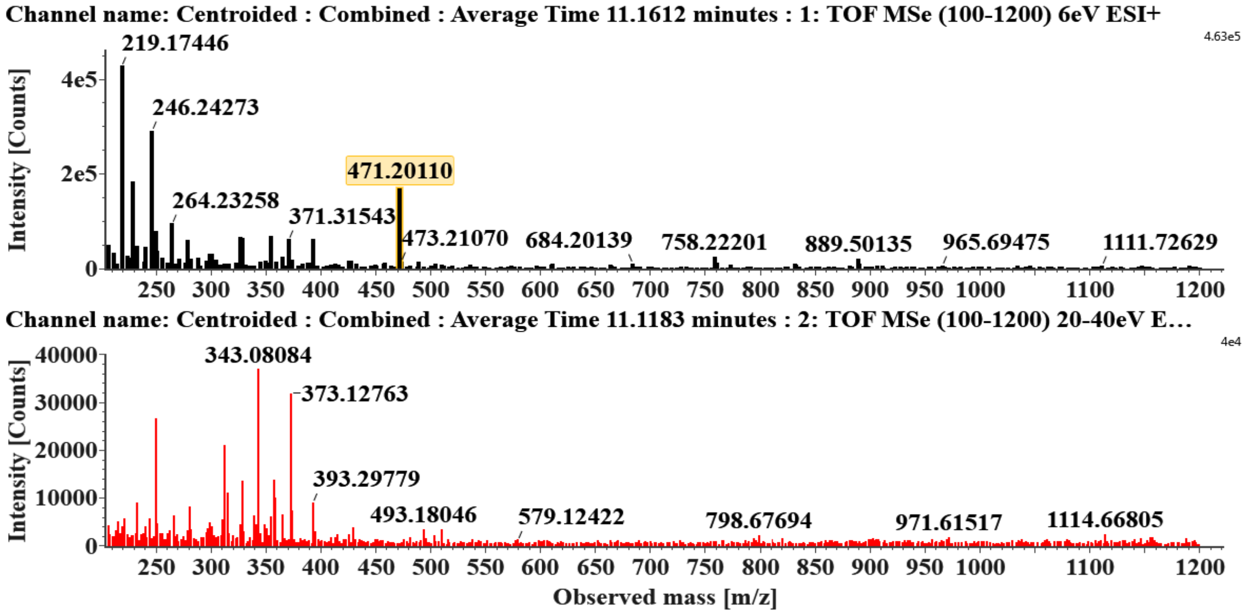

Figure.S18 The Mass chromatogram of Limonin in positive mode: Low energy (Top), High energy (Below).

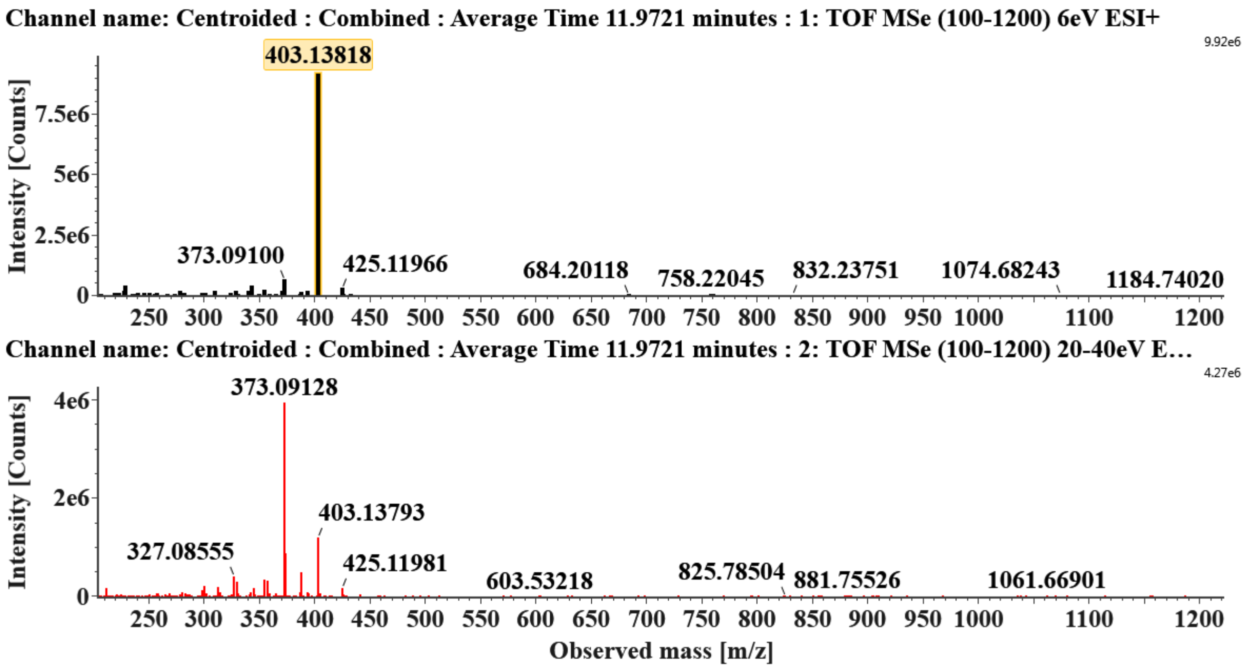

Figure.S19 The Mass chromatogram of 5,6,7,8,3',4'-Hexamethoxyflavone (Nobiletin) in positive mode: Low energy (Top), High energy (Below).

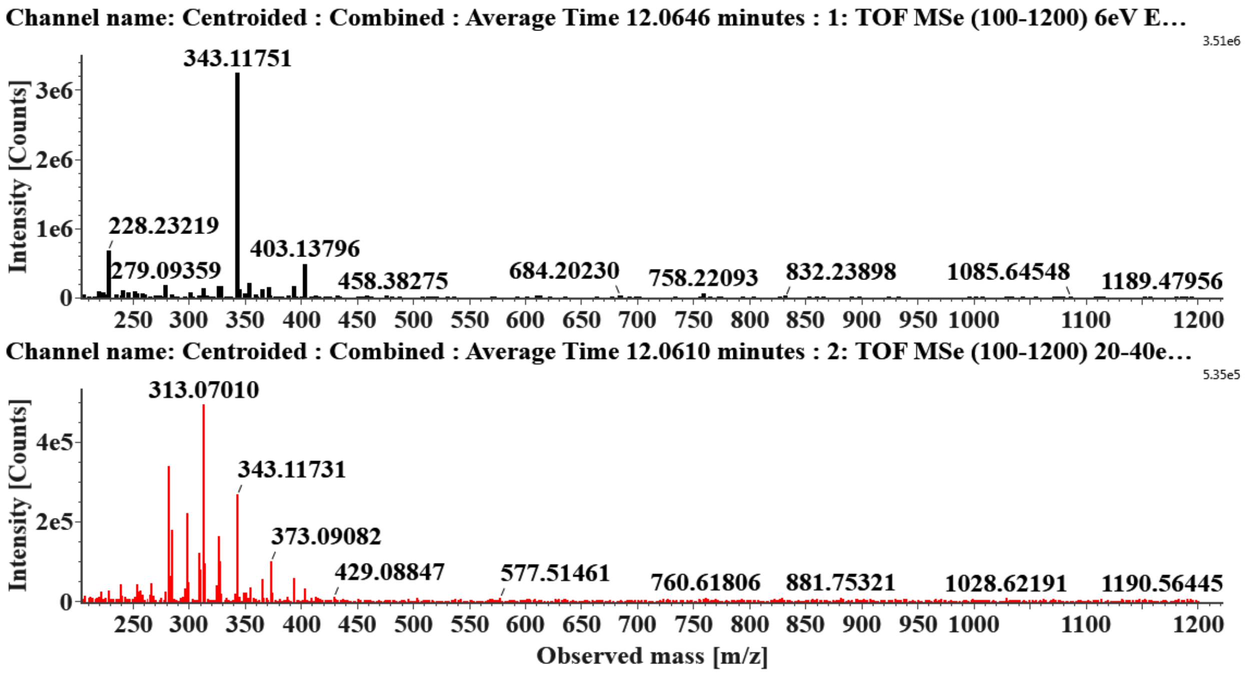

Figure.S20 The Mass chromatogram of 5,7,8,4'-Tetramethoxyflavone in positive mode: Low energy (Top), High energy (Below).

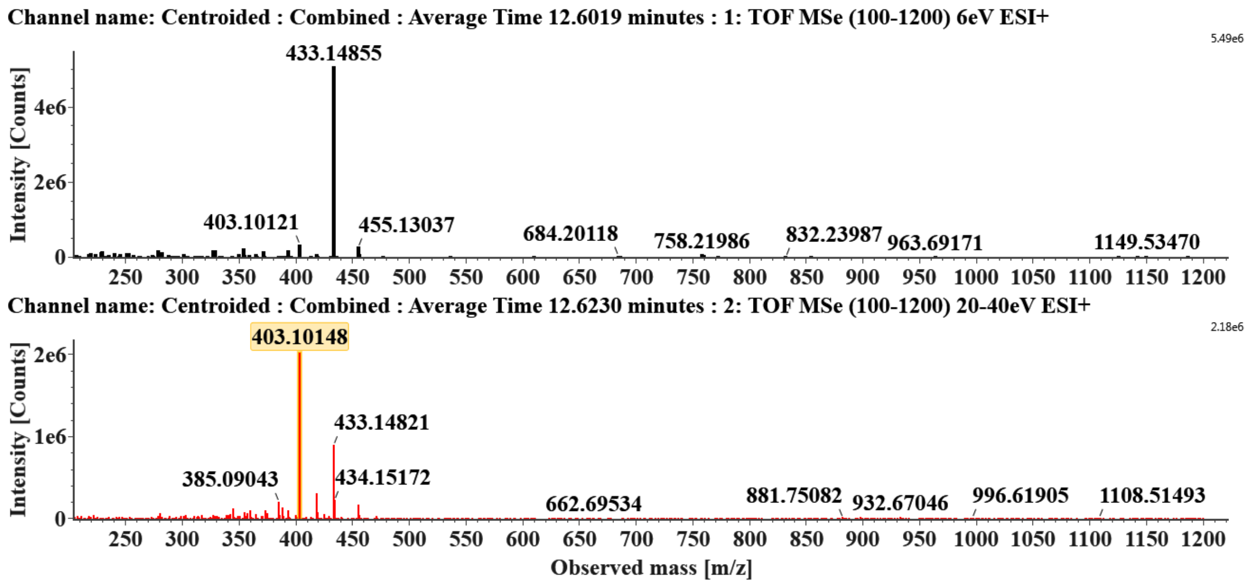

Figure.S21 The Mass chromatogram of 3,5,6,7,8,3',4'-Heptamethoxyflavone in positive mode: Low energy (Top), High energy (Below).

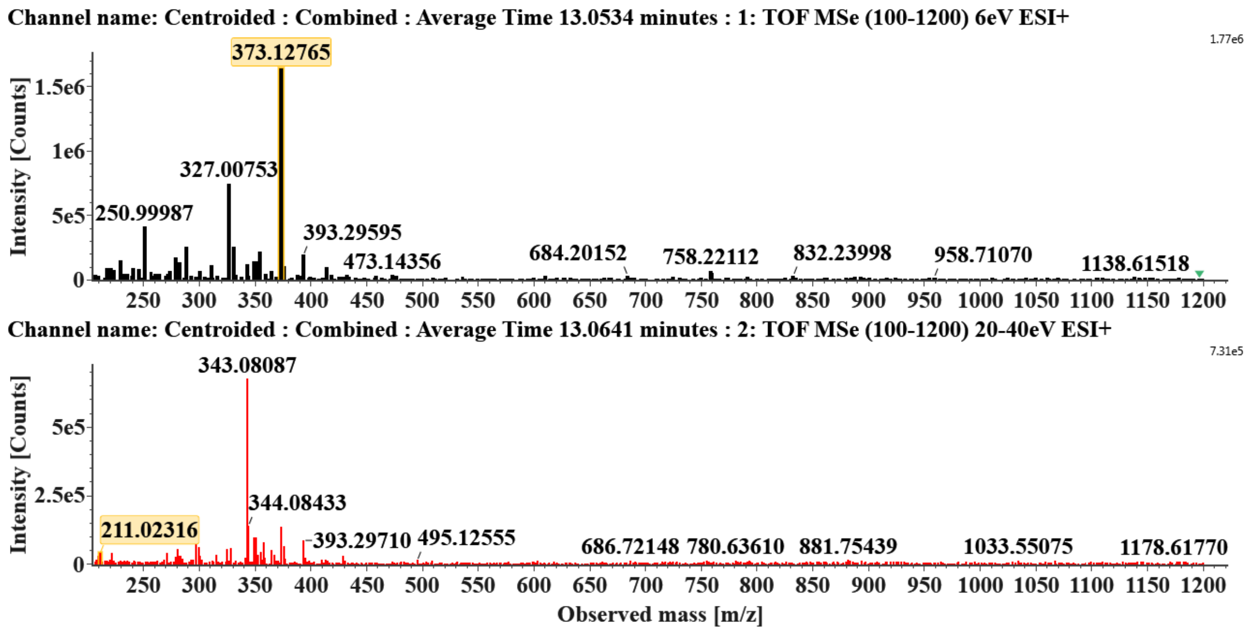

Figure.S22 The Mass chromatogram of 5,6,7,8,4'-Pentamethoxyflavone (Tangeretin) in positive mode: Low energy (Top), High energy (Below).

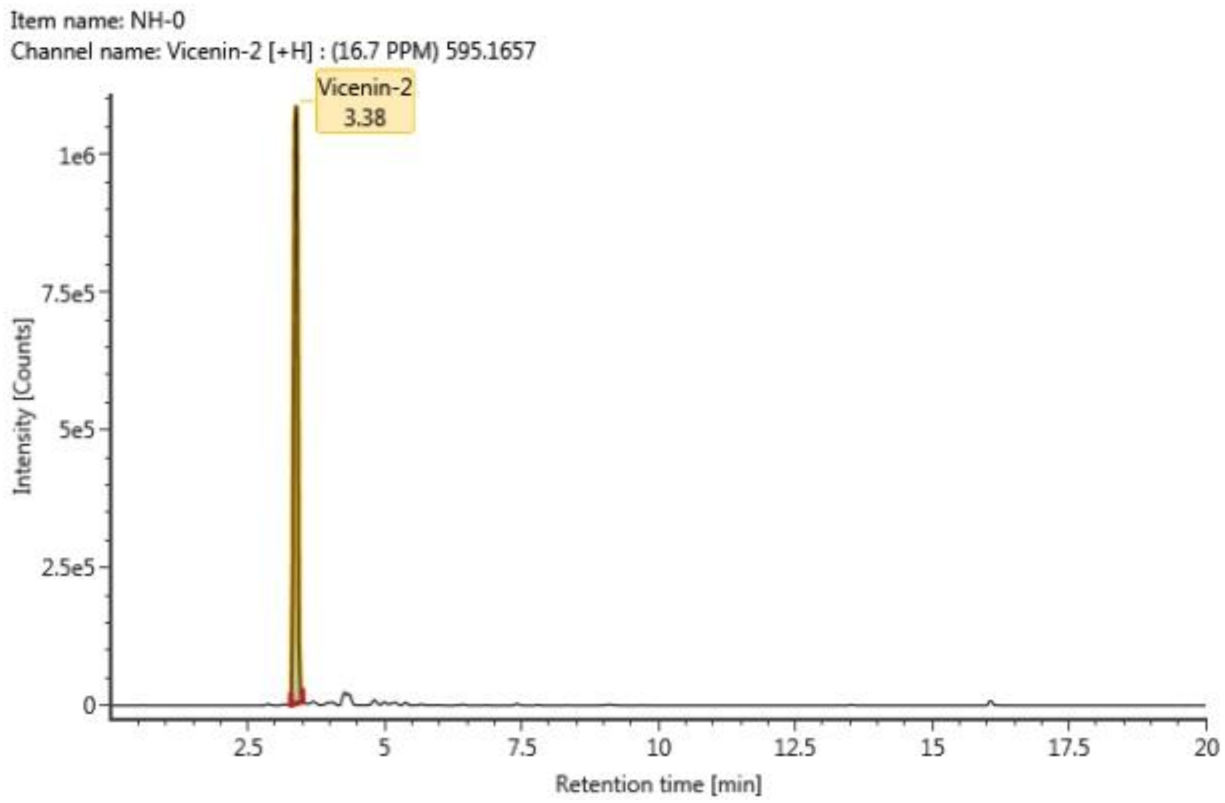

Figure S23. The ESI peak of Apigenin-6,8-di-C-glucoside (Vicenin-2) at 3.38min retention time.

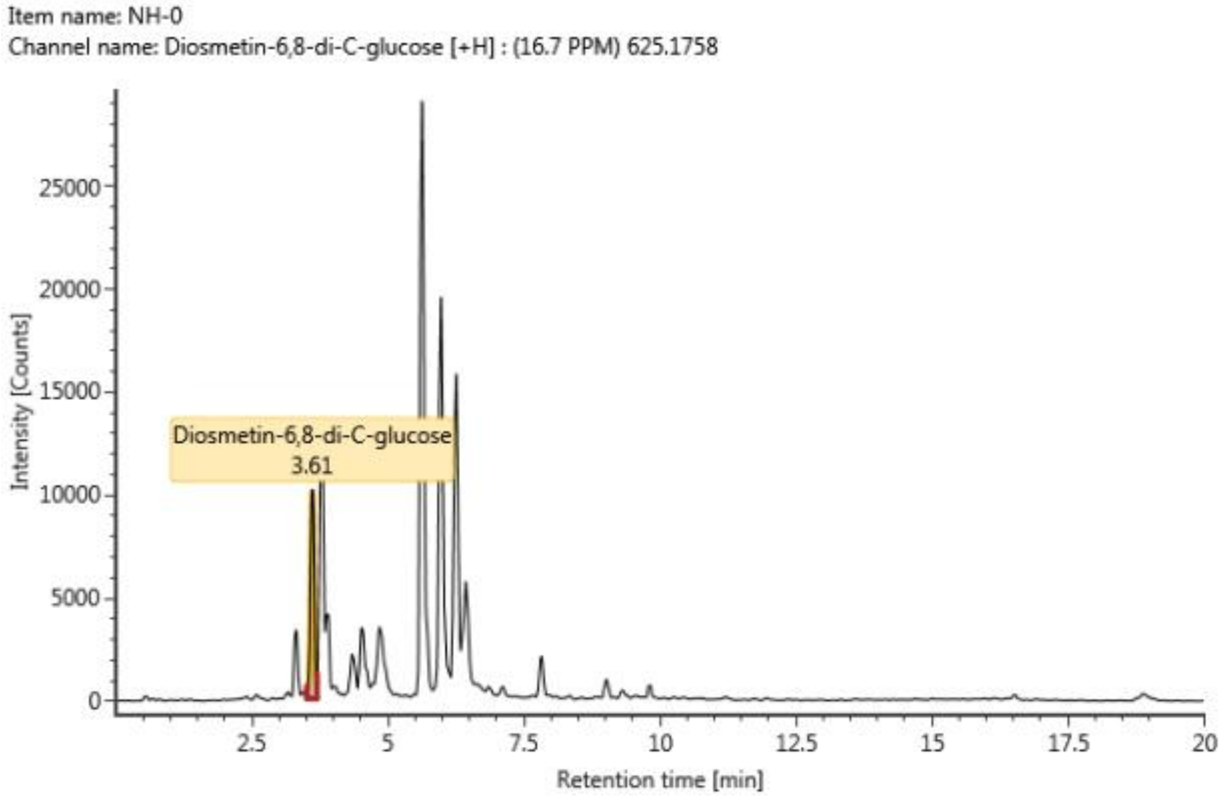

Figure S24. The ESI peak of Diosmetin 6,8-di-C-glucoside at 3.61min retention time.

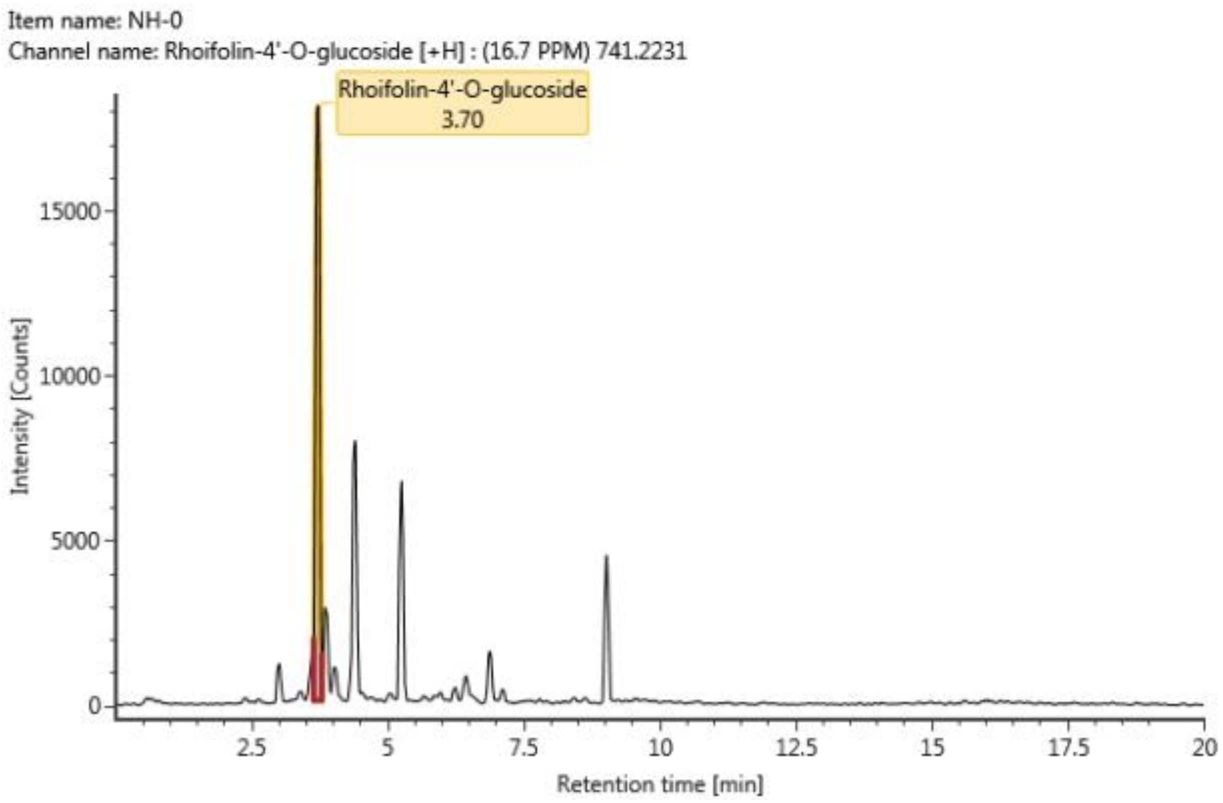

Figure S25. The ESI peak of Rhoifolin-4’-O-glucoside at 3.70min retention time.

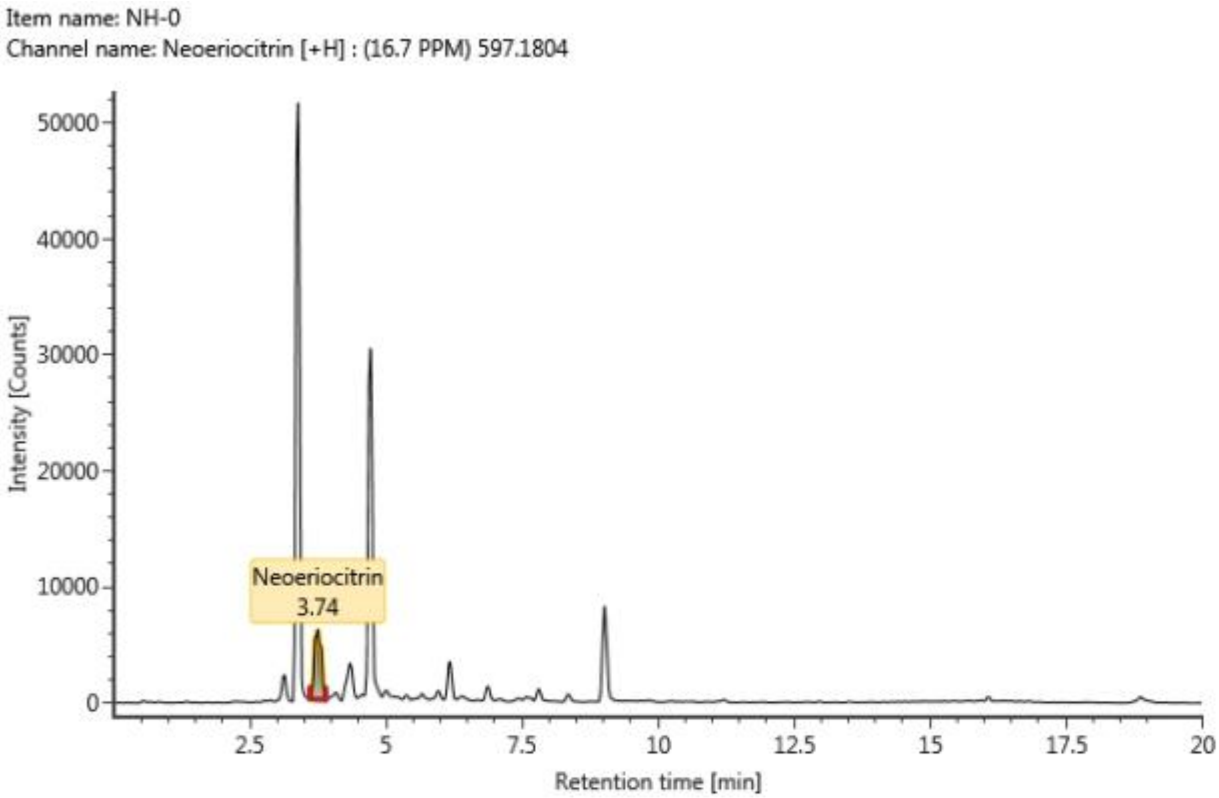

Figure S26. The ESI peak of Neoeriocitrin at 3.74min retention time.

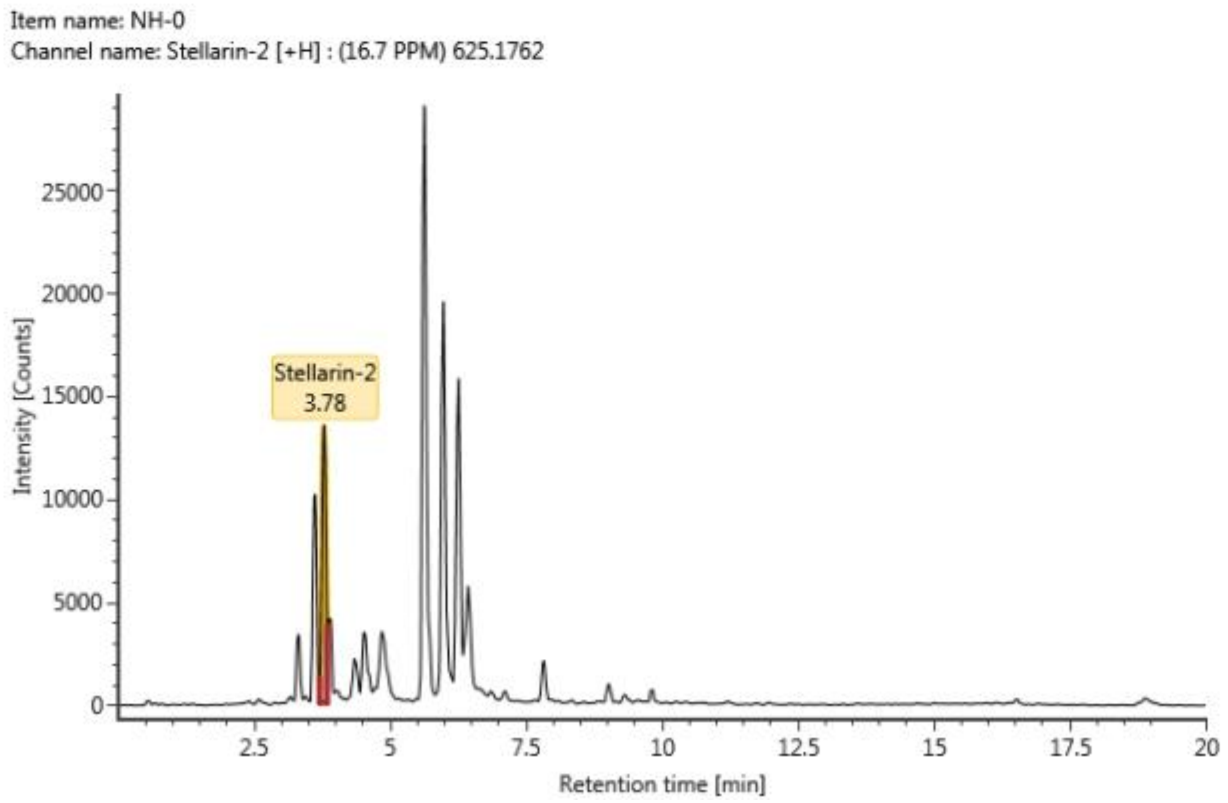

Figure S27. The ESI peak of Chysoeriol-6,8-di-C-glucoside (Stellarin-2) at 3.78min retention time.

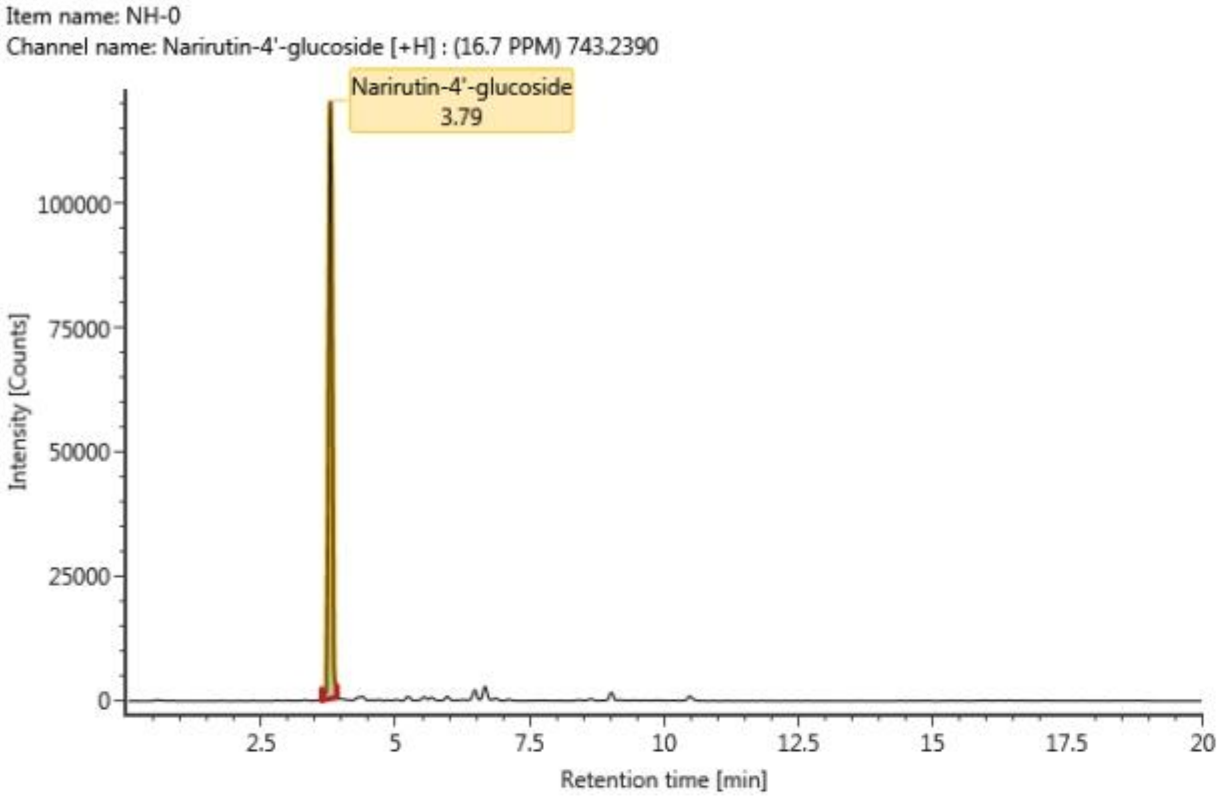

Figure S28. The ESI peak of Narirutin-4’-glucoside at 3.79min retention time.

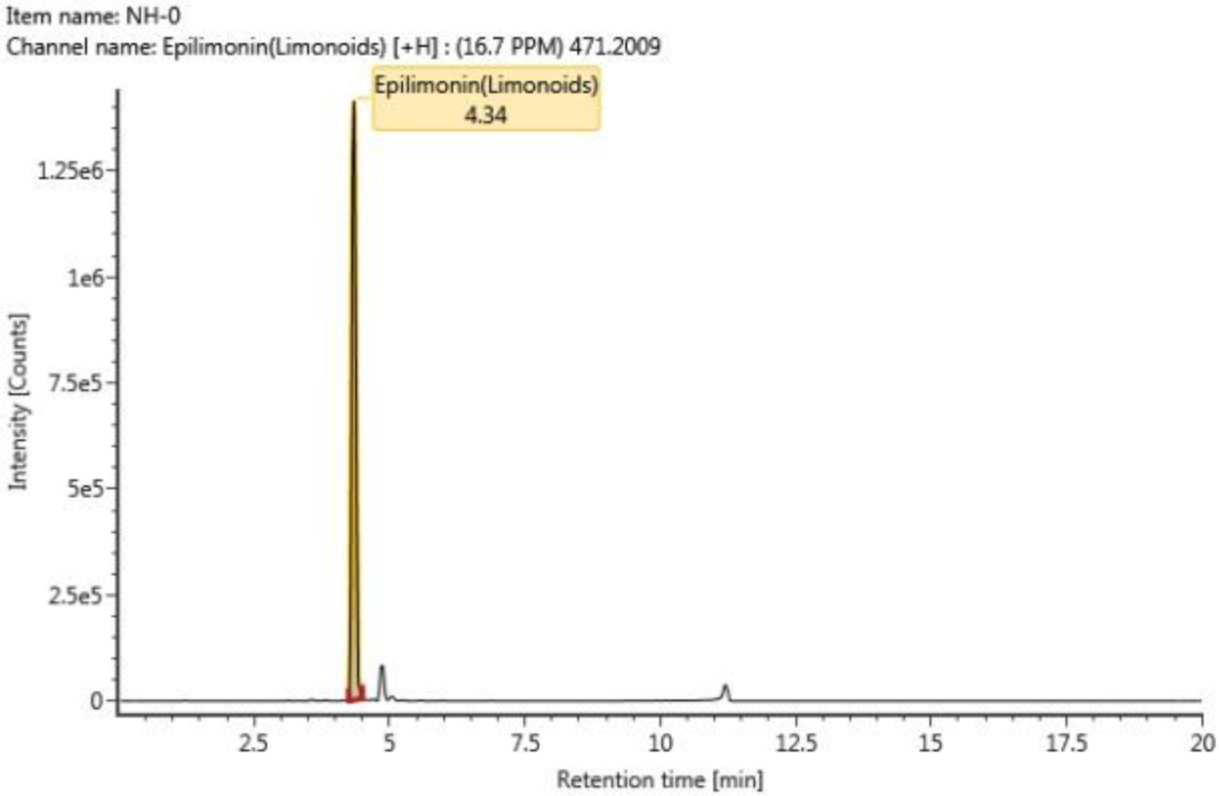

Figure S29. The ESI peak of Epilimonin at 4.34min retention time.

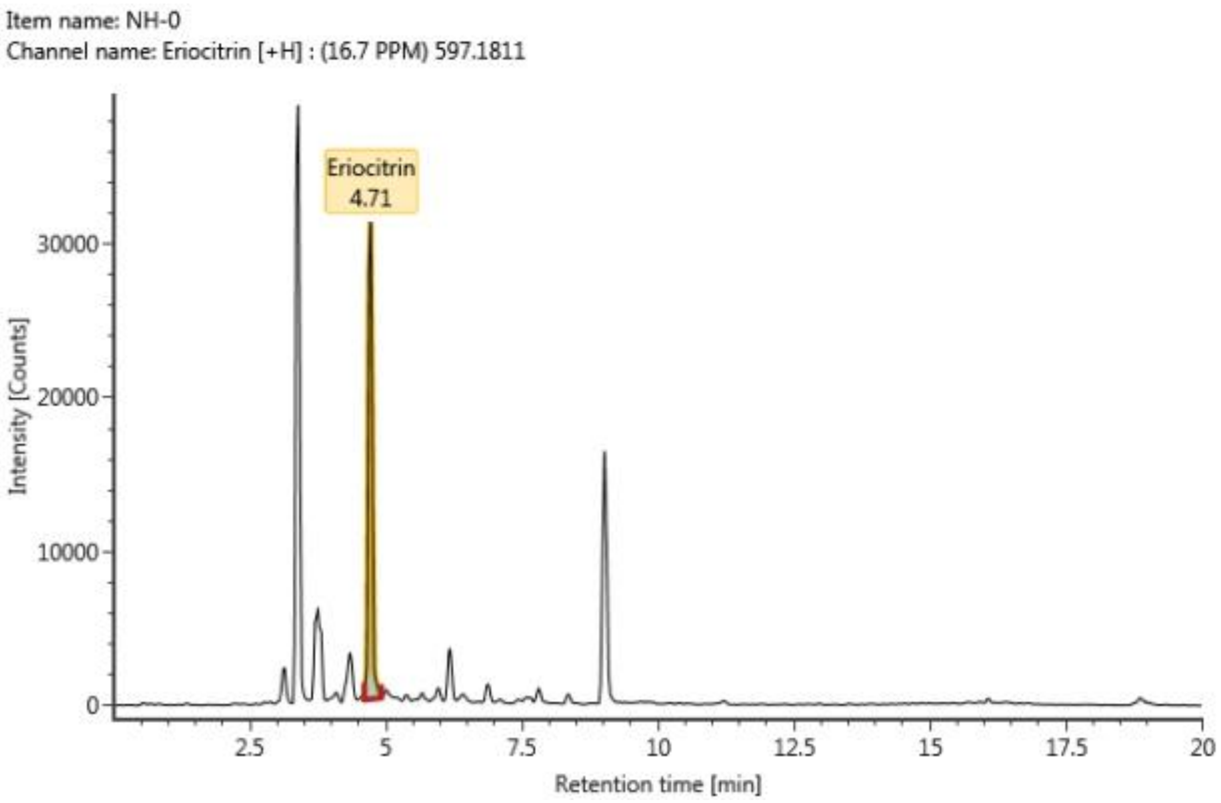

Figure S30. The ESI peak of Eriocitrin at 4.71min retention time.

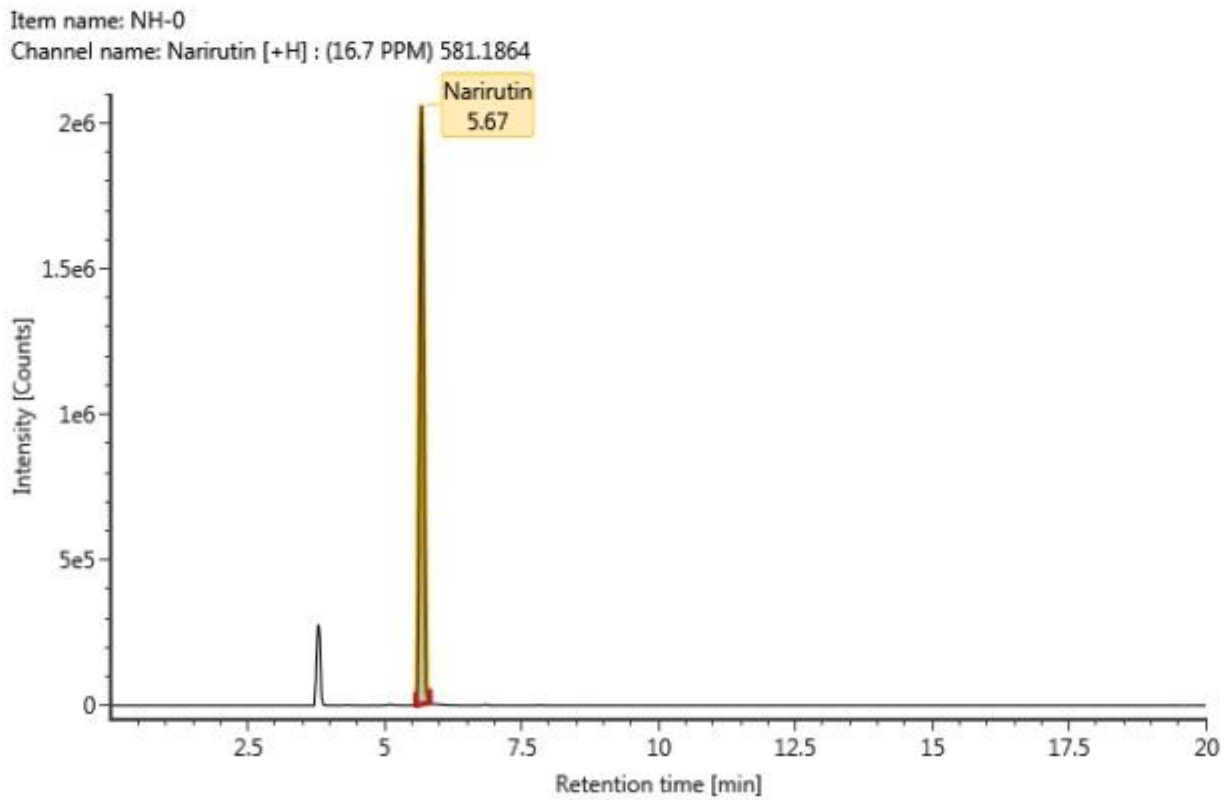

Figure S31. The ESI peak of Narirutin at 5.67min retention time.

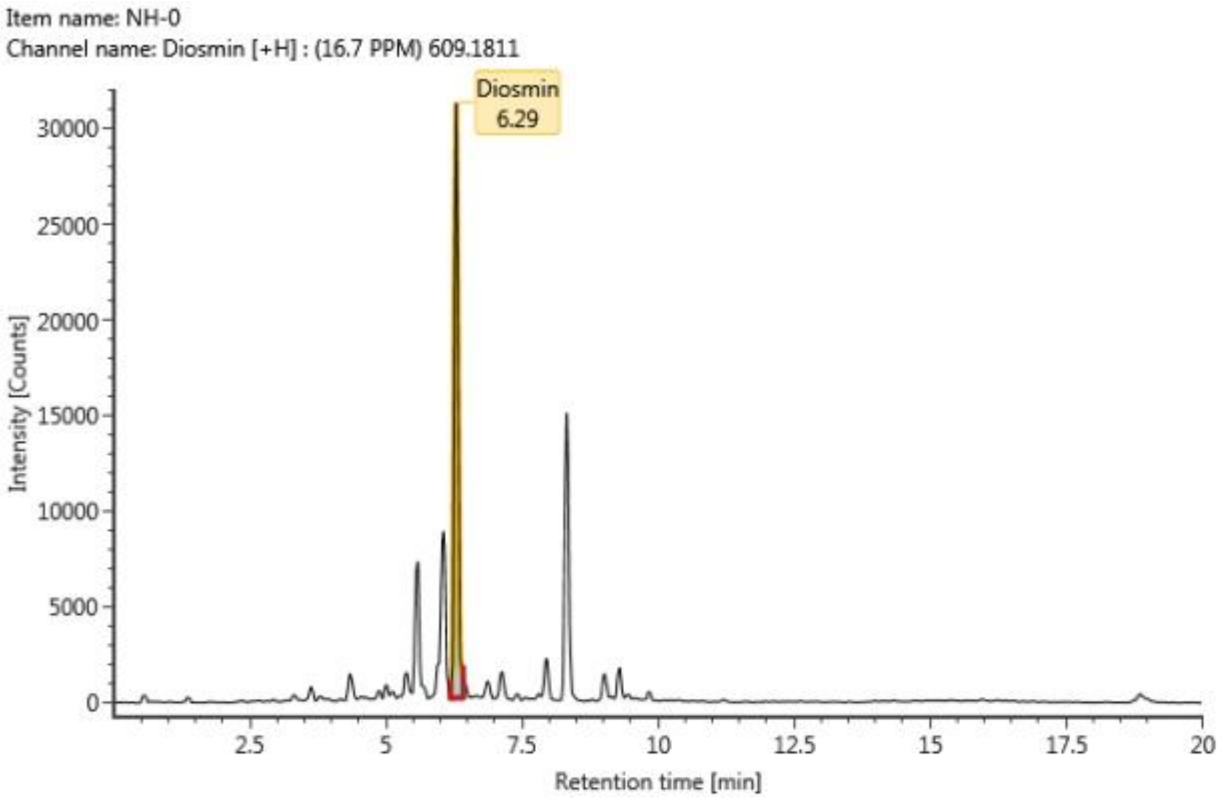

Figure S32. The ESI peak of Diosmin at 6.29min retention time.

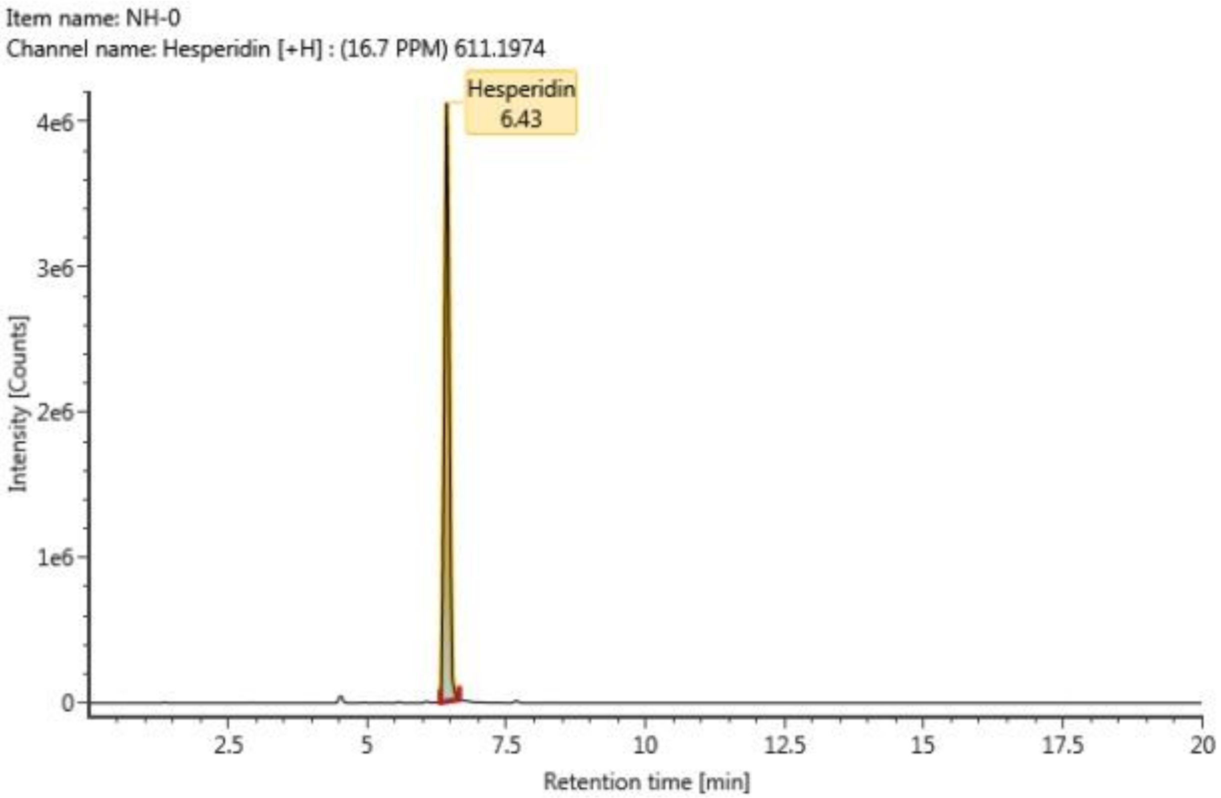

Figure S33. The ESI peak of Hesperidin at 6.43min retention time.

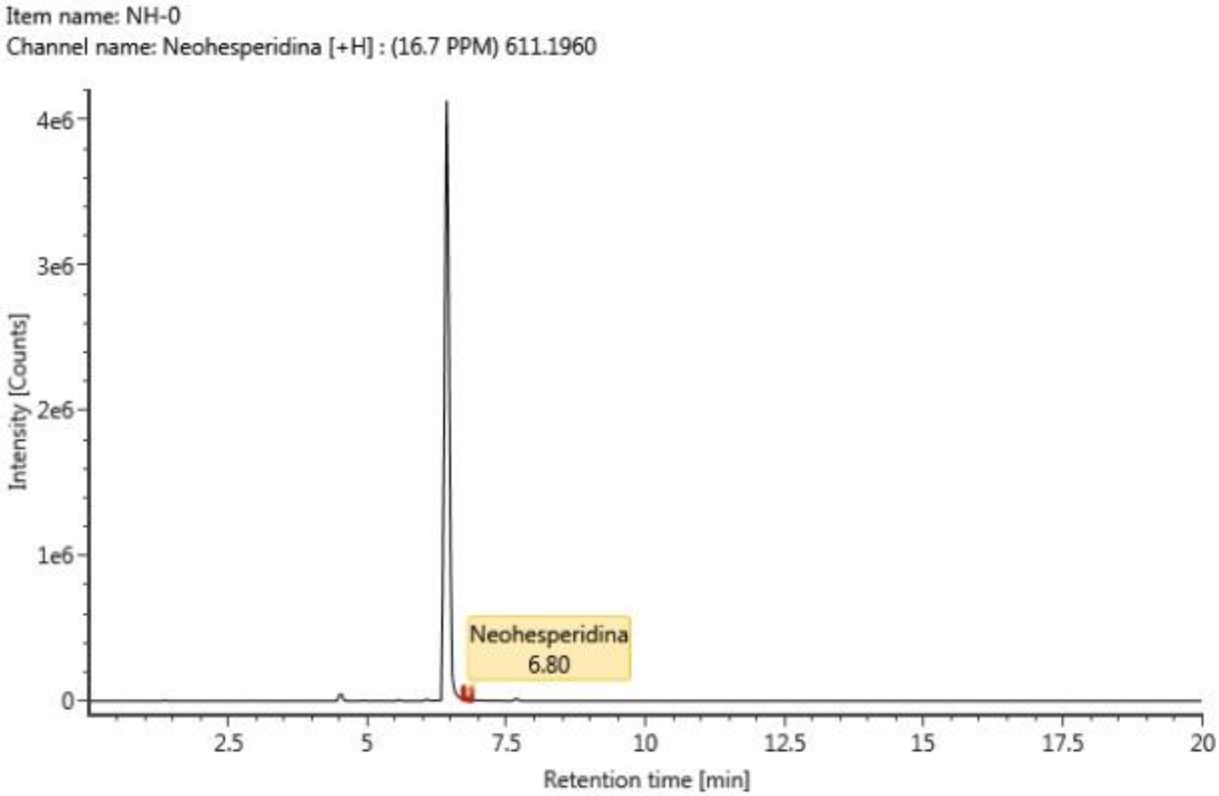

Figure S34. The ESI peak of Neohesperidin at 6.80min retention time.

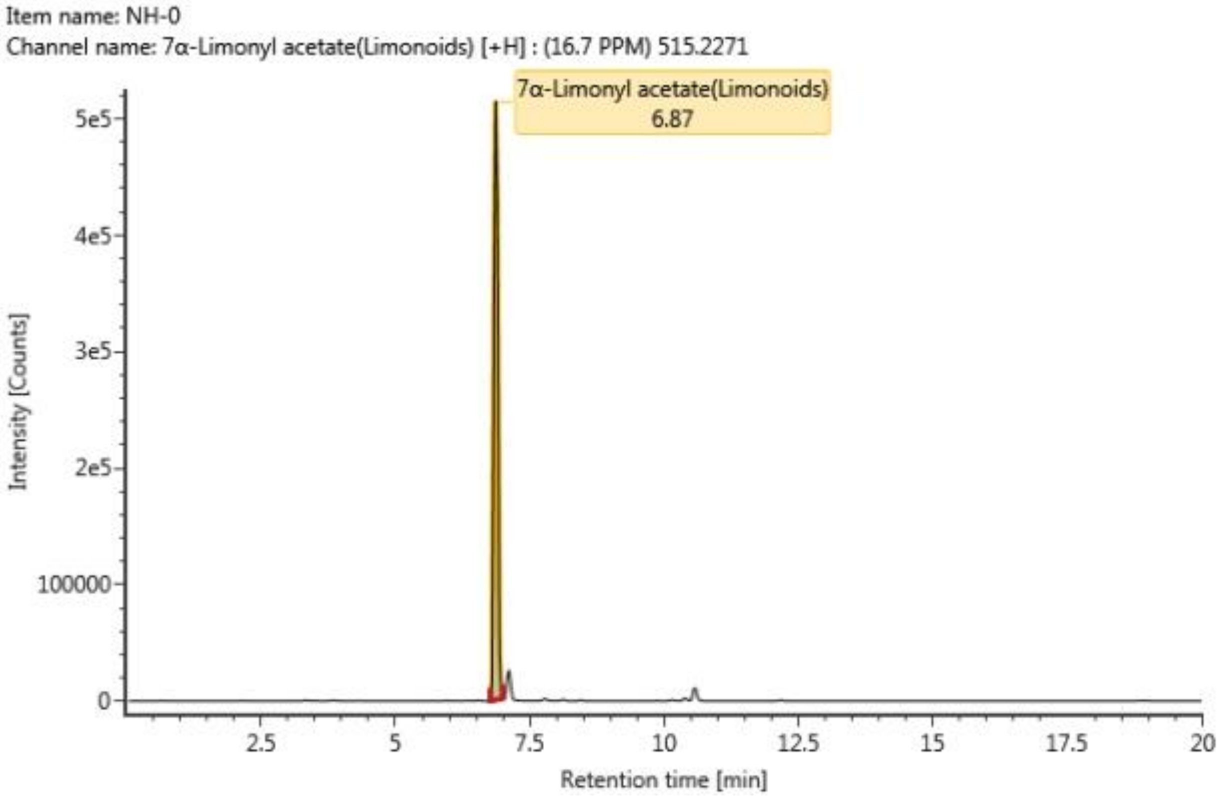

Figure S35. The ESI peak of 7a-Limonyl acetate at 6.87min retention time.

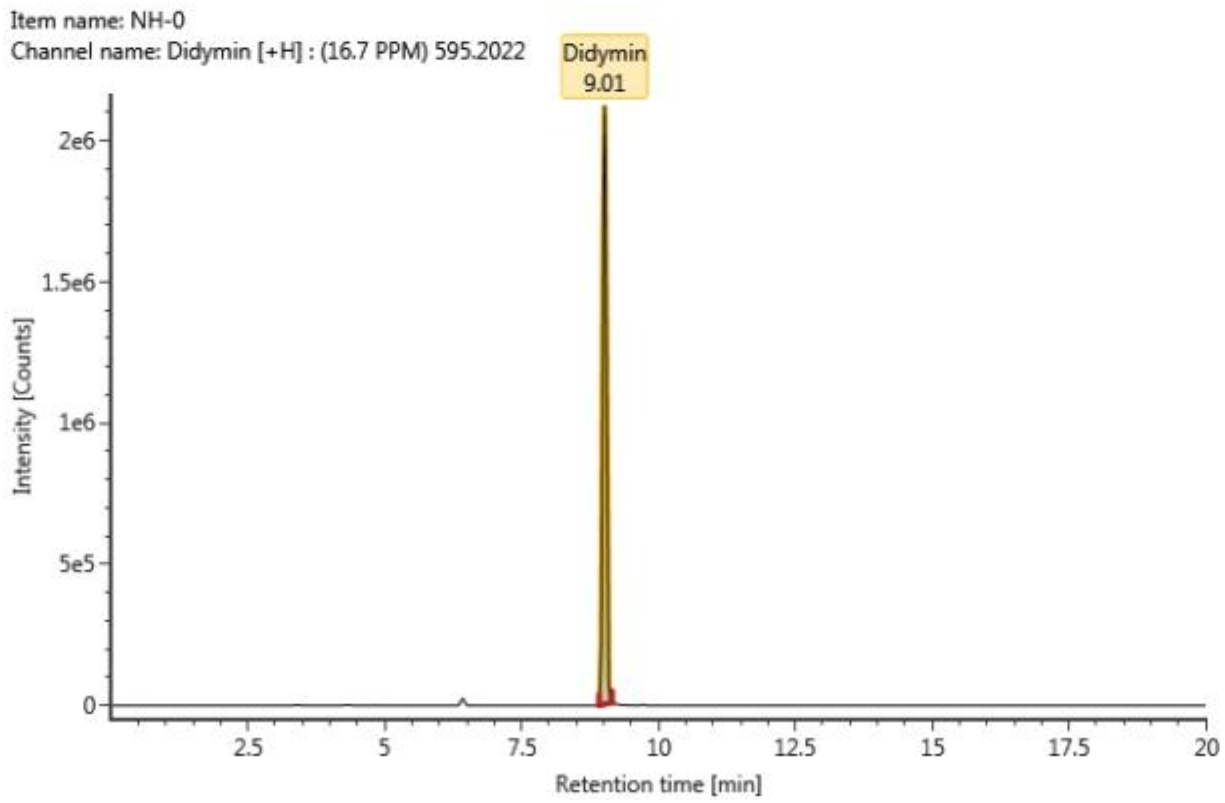

Figure S36. The ESI peak of Didymin at 9.01min retention time.

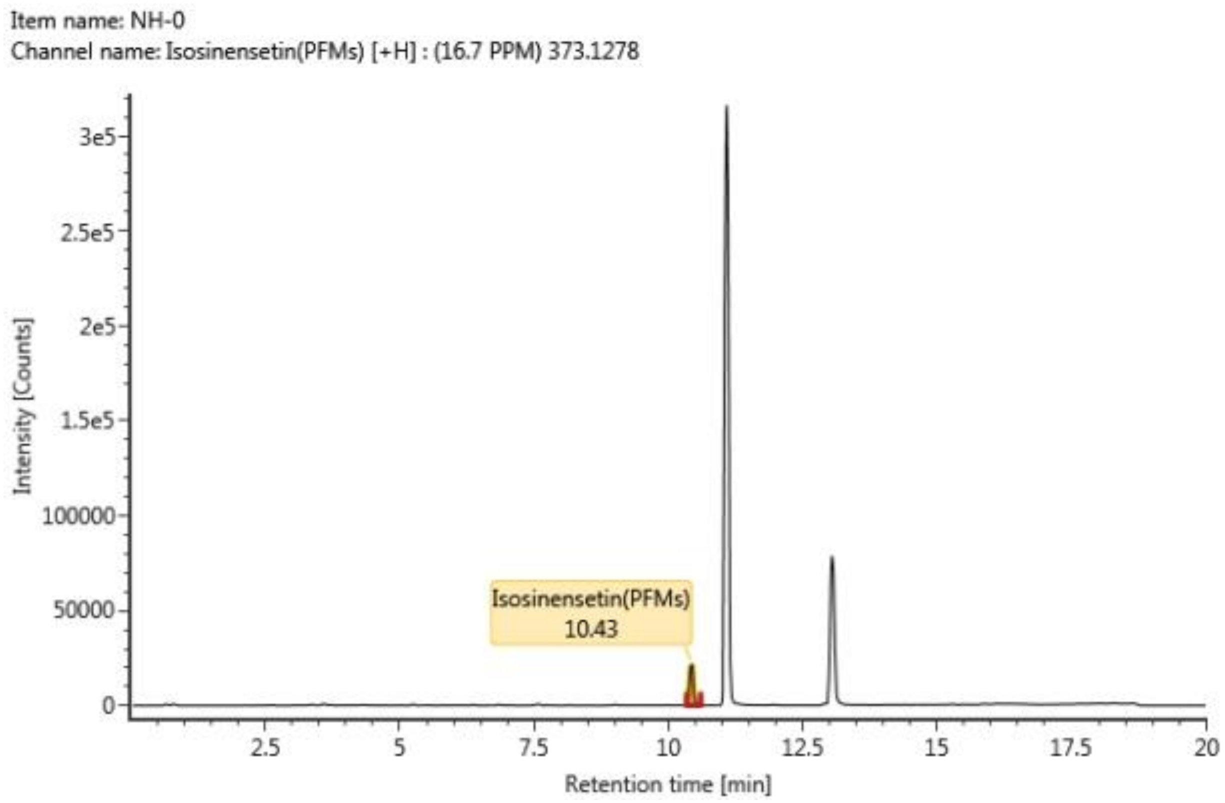

Figure S37. The ESI peak of 5,7,8,3',4'-Pentamethoxyflavone (Isosinensetin) at 10.43min retention time.

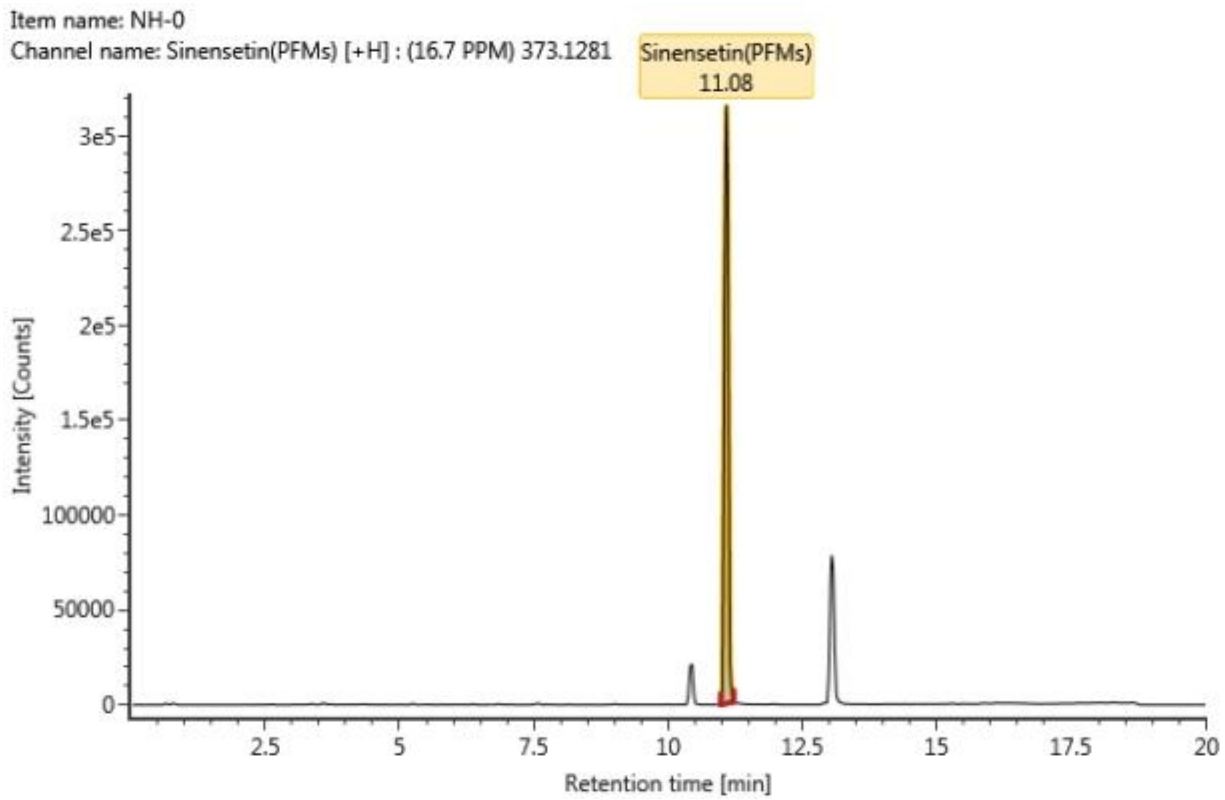

Figure S38. The ESI peak of 5,6,7,3',4'-Pentamethoxyflavone (Sinensetin) at 11.08min retention time.

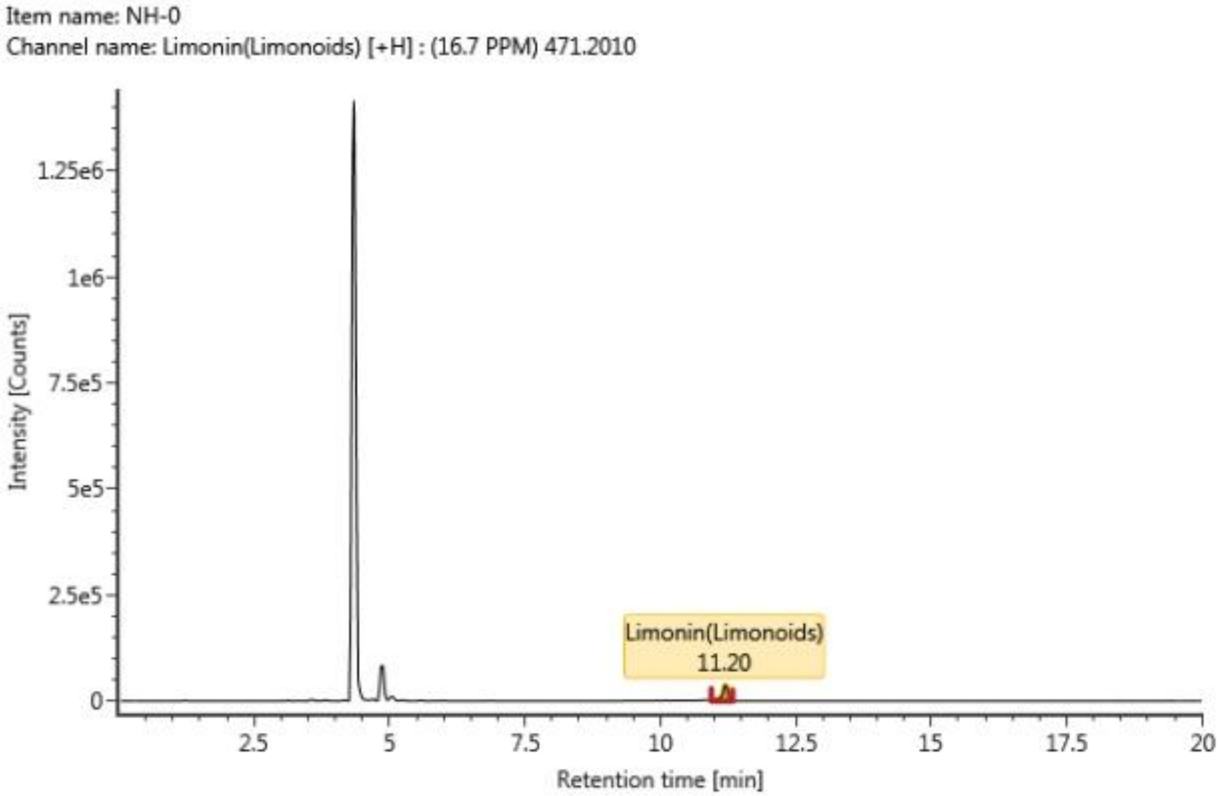

Figure S39. The ESI peak of Limonin at 11.20min retention time.

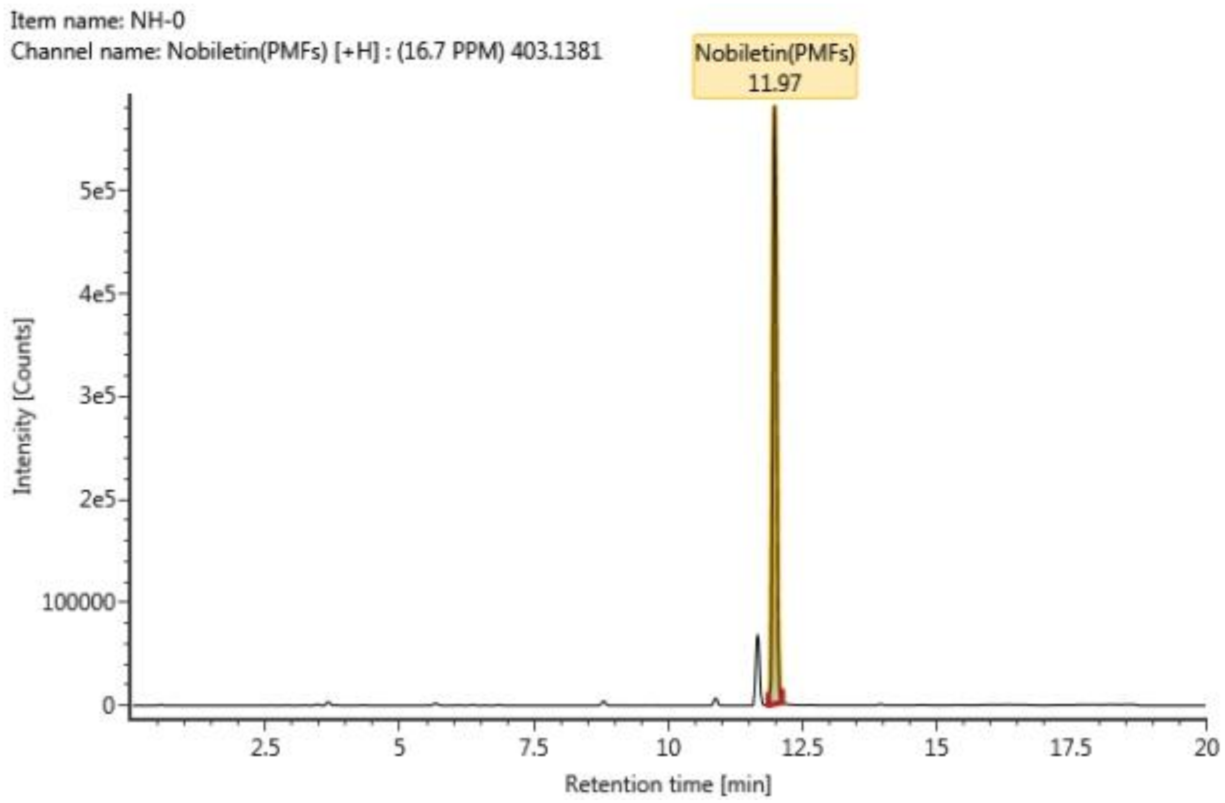

Figure S40. The ESI peak of 5,6,7,8,3',4'-Hexamethoxyflavone (Nobiletin) at 11.97min retention time.

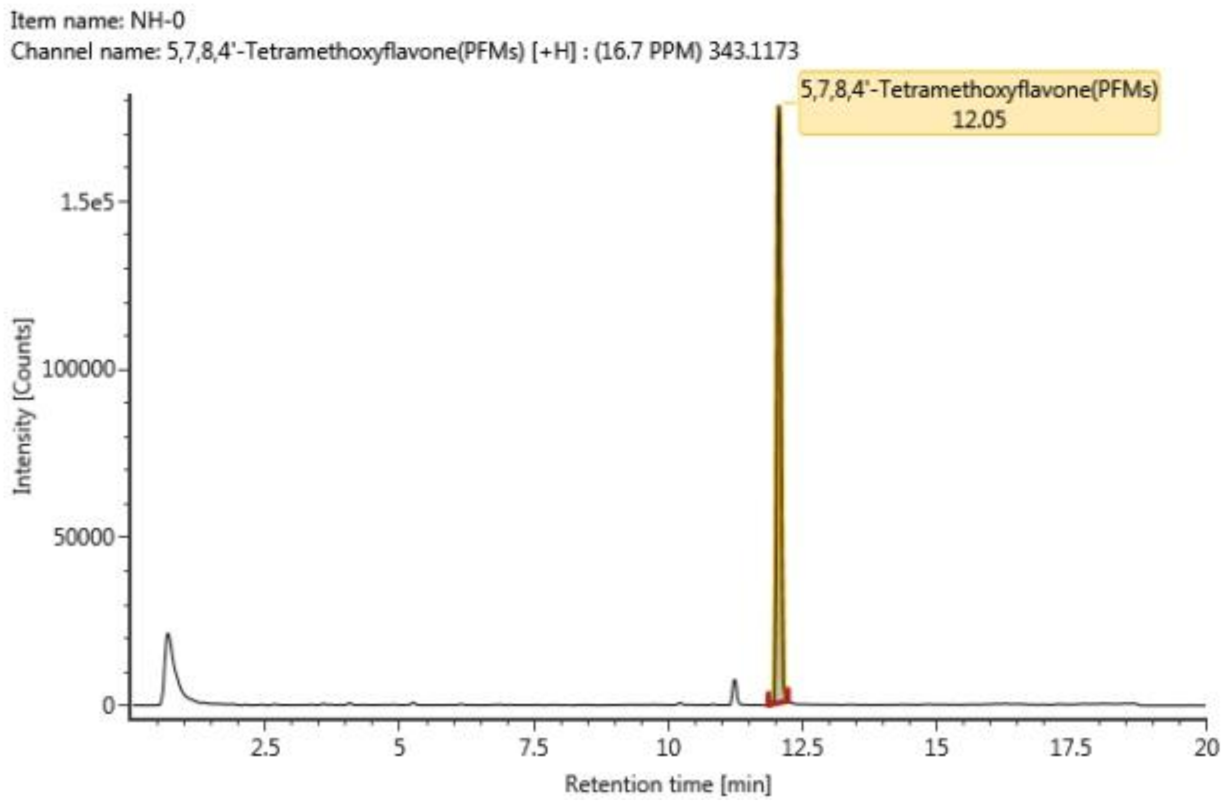

Figure S41. The ESI peak of 5,7,8,4'-Tetramethoxyflavone at 12.05min retention time.

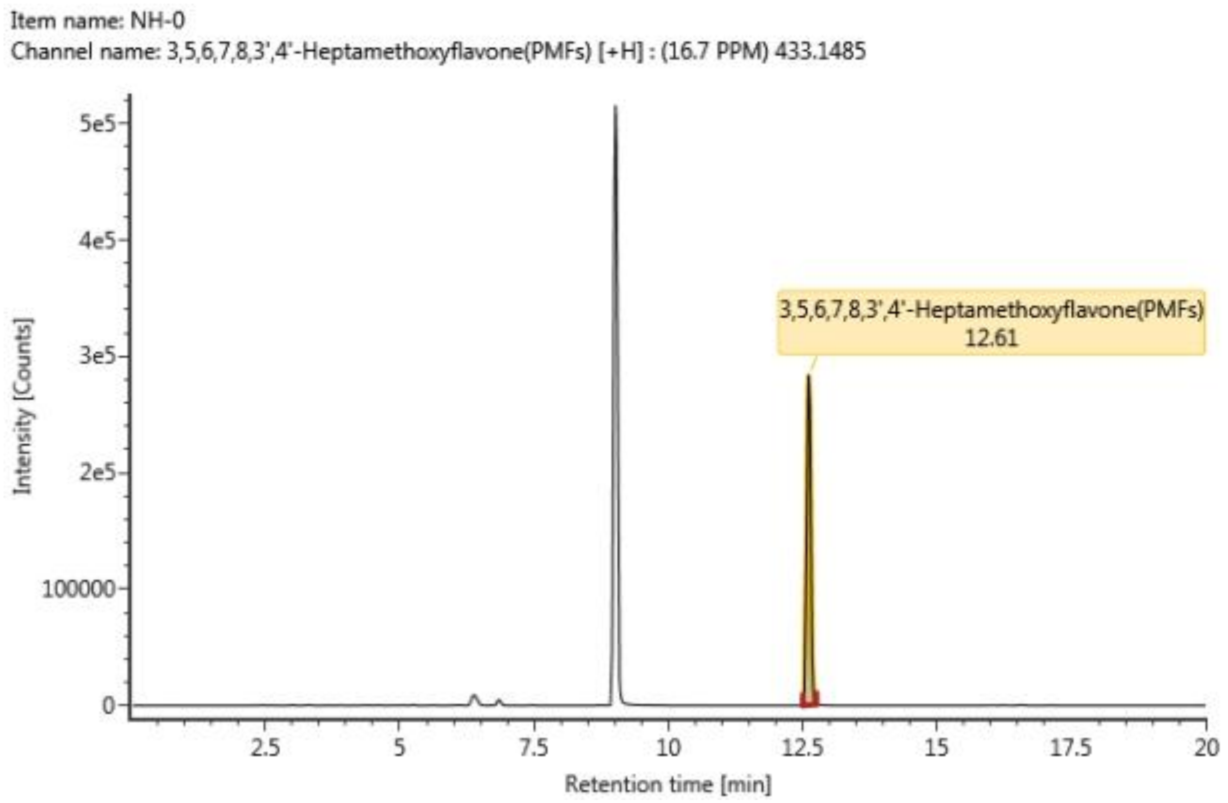

Figure S42. The ESI peak of 3,5,6,7,8,3',4'-Heptamethoxyflavone at 12.61min retention time.

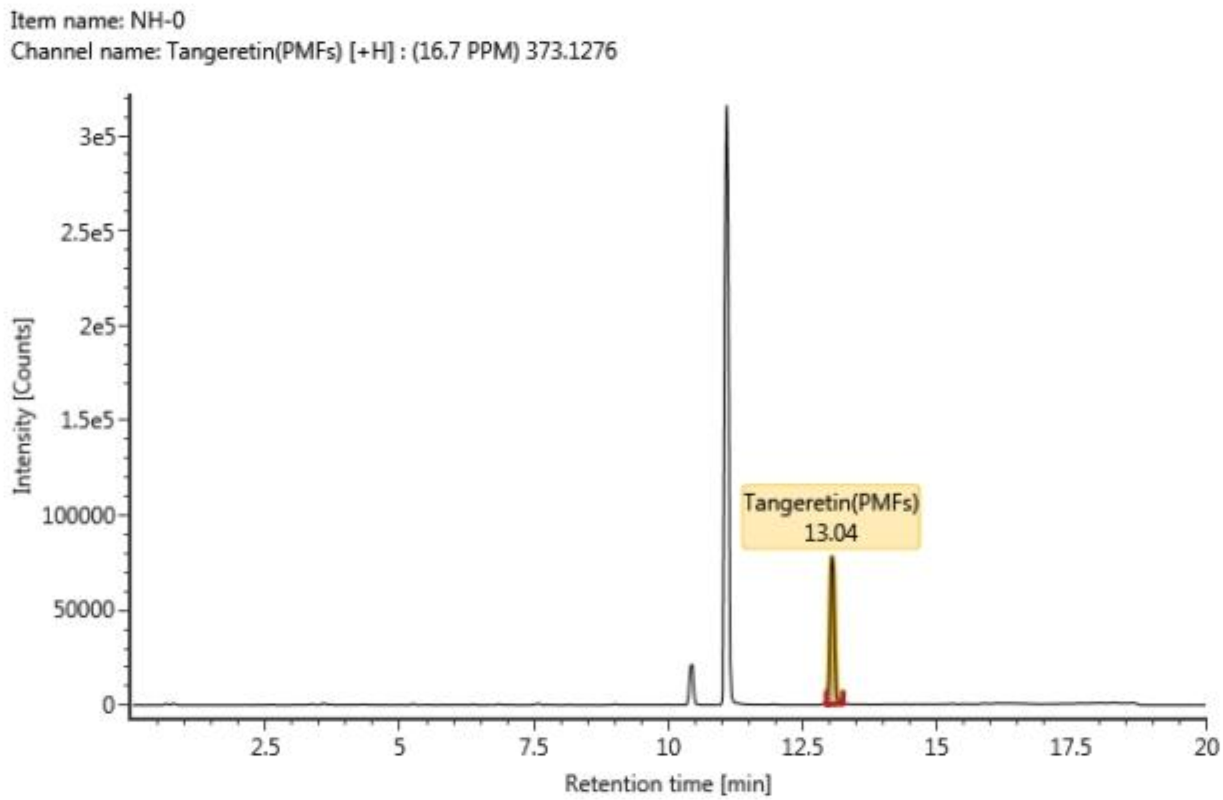

Figure S43. The ESI peak of 5,6,7,8,4'-Pentamethoxyflavone (Tangeretin) at 13.04min retention time.
